# The RHNumtS compilation: Features and bioinformatics approaches to locate and quantify Human NumtS

**DOI:** 10.1186/1471-2164-9-267

**Published:** 2008-06-03

**Authors:** Daniela Lascaro, Stefano Castellana, Giuseppe Gasparre, Giovanni Romeo, Cecilia Saccone, Marcella Attimonelli

**Affiliations:** 1 Dipartimento di Biochimica e Biologia Molecolare "E. Quagliariello", Università di Bari, Via E. Orabona 4, 70126 Bari, Italy; 2 Unità di Genetica Medica, Policlinico Universitario S. Orsola-Malpighi, Università di Bologna, 40138 Bologna, Italy

## Abstract

**Background:**

To a greater or lesser extent, eukaryotic nuclear genomes contain fragments of their mitochondrial genome counterpart, deriving from the random insertion of damaged mtDNA fragments. NumtS (Nuclear mt Sequences) are not equally abundant in all species, and are redundant and polymorphic in terms of copy number. In population and clinical genetics, it is important to have a complete overview of NumtS quantity and location. Searching PubMed for NumtS or Mitochondrial pseudo-genes yields hundreds of papers reporting Human NumtS compilations produced by *in silico *or wet-lab approaches. A comparison of published compilations clearly shows significant discrepancies among data, due both to unwise application of Bioinformatics methods and to a not yet correctly assembled nuclear genome. To optimize quantification and location of NumtS, we produced a consensus compilation of Human NumtS by applying various bioinformatics approaches.

**Results:**

Location and quantification of NumtS may be achieved by applying database similarity searching methods: we have applied various methods such as Blastn, MegaBlast and BLAT, changing both parameters and database; the results were compared, further analysed and checked against the already published compilations, thus producing the Reference Human Numt Sequences (RHNumtS) compilation. The resulting NumtS total 190.

**Conclusion:**

The RHNumtS compilation represents a highly reliable reference basis, which may allow designing a lab protocol to test the actual existence of each NumtS. Here we report preliminary results based on PCR amplification and sequencing on 41 NumtS selected from RHNumtS among those with lower score. In parallel, we are currently designing the RHNumtS database structure for implementation in the HmtDB resource. In the future, the same database will host NumtS compilations from other organisms, but these will be generated only when the nuclear genome of a specific organism has reached a high-quality level of assembly.

## Background

In greater or lesser abundance, eukaryotic nuclear genomes contain fragments of their mitochondrial (mt) genome counterpart, deriving from "random" insertion of damaged mtDNA fragments [1]. The discovery of these genomic "elements" dates back to 1967, when du Buy and Riley [2] discovered mtDNA sequences in the nuclear genome by means of hybridization experiments on mouse liver. The presence of mtDNA in the nuclear genome was confirmed in 1983, in yeast, locust, fungi, sea urchin, man, maize and rat [3-9]. In 1994 Lopez et al. [10] called these fragments *numt*, in this paper renamed NumtS, Nuclear mt Sequences. One hypothesis on the mechanism of their generation suggests that fragments of mtDNA may escape from mitochondria to avoid mutagenic agents or other forms of cellular stress, reach the nucleus and, during repair of chromosomal breaks, insert into the nuclear DNA [11]. Papers published so far report that NumtS loci do not show a common feature at integration sites [12]. The NumtS generation process may have started soon after endosymbiosis. It seems obvious that the genomic region where the mt sequence is inserted may be involved in further recombination events, thus generating duplication of the mt fragment. In some organisms, such as primates, the same mt region occurs several times along the nuclear genome, but only detailed evolutionary analysis may help in identifying "duplicated" NumtS, because recombination and mutation occurring after duplication may well mask the latter event. Once this problem is solved, each NumtS may be associated with a given copy number, although this may differ even among tissues or cells of the same individual. NumtS have been shown in fact to be polymorphic: a specific NumtS may be present in heterozygosis in the same individual or may be totally absent in a specific tissue or individual. The first evidence of the polymorphic nature of NumtS was reported by Zischler et al. [13], in which an insert of 540 bp (reverse positions (59–16089) of the revised Cambridge Reference Sequence (rCRS) [14]), located on chromosome 11 and detected on total DNA extracted from sperm, was screened in various populations using primers designed on the sequences flanking the insertion. Among the screened individuals, some were homozygous, some were heterozygous and some did not show the insertion. When present, the inserted sequence was highly conserved in all populations, thus revealing a "nuclear fossil". The inserted sequence with its nuclear flanking region is available through GenBank entry S80333 but, when this sequence is blasted against Human nuclear genome Build 36.2, the resulting hit matches only the flanking region. This means that the samples used for sequencing the Human Genome did not harbour the "insertion". Further examples of polymorphic NumtS are reported in "The case of siblings" [15] and the Ricchetti compilation [16].

**Figure 1:**
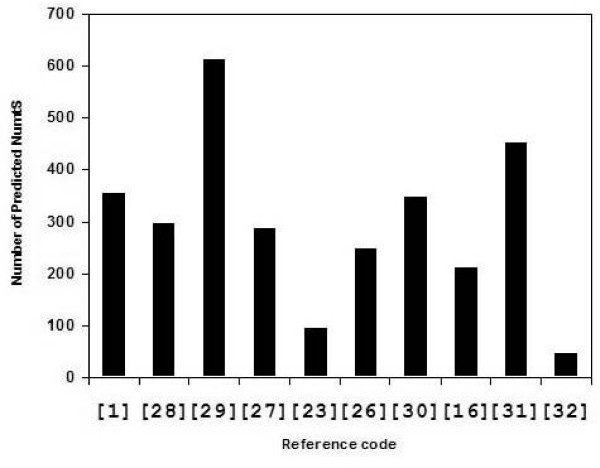
**Number of NumtS reported in a selected group of published compilations**. Horizontal axis reports the reference number in this paper of the analysed compilation.

NumtS are not equally abundant in all species. For instance a much higher number of NumtS occurs in plants with respect to Metazoa. Within Metazoan, NumtS are more abundant in mammals and birds, but a very small number can be found in *Plasmodium*, *Caenorhabditis *and *Drosophila*. The debate about the presence or absence of NumtS in *fugu *is open [17,18]. The great abundance of NumtS in *Apis mellifera*, comparable to that in plants, has been published recently [19]. A complete knowledge of Human NumtS is of fundamental importance in the study of human population migrations, which utilize mtDNA as a phylogenetic marker, and also in the study of mitochondrial diseases. NumtS are in fact a potential source of contamination when PCR is used to study mtDNA. This is particularly important in the case of ancient DNA or tissue with a reduced quantity of mtDNA copy number, in both physiological (sperm) and pathological states [20,21]. Bensasson et al. [1] presented an exhaustive *vademecum *suggesting how to check and avoid NumtS contamination. A final consideration concerns the observation that since NumtS reside in the nucleus, they should evolve much more slowly than their functional counterparts in the nucleus, so that they represent nuclear fossils, "snapshots" of mtDNA at the time of transfer. This allows them to be used as outgroups in phylogenetic studies [22-25]. Searching PubMed for NumtS or Mitochondrial pseudogenes in November 2006 yielded hundreds of papers, 113 of which on humans. Many of them report the compilation of Human NumtS and other Eukaryotic Genomes [1,16,23,26-31], and usually mention the location and length of each NumtS. Such data were mainly obtained by *in silico *approaches and only a minority derived from a wet-lab approach, sequencing or nDNA-mtDNA hybridizations [32,16]. Parr et al. [32] demonstrated by sequencing that the entire mitochondrial genome is present within the nuclear genome in multiple copies: the "pseudo-mitochondrial genome". A comparison of published compilations highlights great discrepancies among data (Figure 1). The reason for this lies in two important facts: incautious usage of Bioinformatics methods and application of methods to a still not yet correctly assembled nuclear genome. However, the trends common to all papers are that the number of NumtS varies among species and that the human genome apparently contains the highest number of NumtS within Metazoan. But the data are still incomplete and imperfect. NumtS quantification needs revision, particularly starting from Human data. The present paper, describing the bioinformatics approaches used to optimize quantification and localization of NumtS, reports the Consensus Reference Human NumtS Compilation (RHNumtS) and the results of the amplification and sequencing approach applied to 41 selected NumtS

## Results

The location and quantification of NumtS may be achieved by applying database similarity searching methods, comparing the human mt DNA sequence with human nuclear genome sequences. The goal of database similarity searching methods is to seek for regions showing statistically meaningful similarity, but these methods are too sensitive: slight changes in parameters and/or in both query and database sequences may give rise to considerable changes in the resulting hits. In this study the available human mt DNA sequences total at present more than 3000, of which about 90% represent different haplotypes, thus choosing the query sequence is the first problem. Moreover available human nuclear genome sequences are consensus sequences, obtained from the DNA of five different individuals, and suffer from physiological assembly limits, due to great repetition in the genome. This means that there are slight differences which may lead to poorly reproducible results, due to mitochondrial polymorphic sites and to a still not completely defined nuclear genome. Thus, changing methods or parameters or applying the method to different human genome sequence collections produce diverse results. Here, we suggest the usage of different approaches and the comparison of the obtained results, in order to minimize both false positive and negative results with the aim to optimize quantification and localization of human NumtS. Our overall proposal is to apply several bioinformatics approaches, to compare the results, to produce a *consensus *compilation and then validate the results through PCR and sequencing analysis. This last step is not the primary scope of this work although we report some preliminary results. Available bioinformatics programs for this type of analysis are BLAST, BLAT and FASTA. We used only BLAST [33,34] and BLAT [35], the performance of which better suited our needs. BLAST is implemented in many versions, Blastn, Blastx, tBLastn, MegaBlast, etc. We used Blastn and MegaBlast, the versions most frequently adopted in reports on human NumtS compilation.

### Blastn results

Tables 1 and 2 summarise the results obtained with Blastn, searching the revised Reference Cambridge Sequence [14] (J01415.2) similarity versus various human nucleotide sequence datasets by changing limits by *Entrez *and limits on number of positive hits to be displayed (see Methods section). The data in the tables clearly show how easy it is to obtain false positive or negative hits. Among the various results, we chose the one producing 2145 hits, comparing the J01415.2 sequence against the Chromosome Genome database with the *Entrez *limit "homo sapiens [ORGN] NOT (mitochondrion OR mitochondrial) [ALL]" since in this case hits on the mitochondrial genome were not obtained (query #19 in Table 2). The data shown here were produced in March 2007, when the old version of Blast was available. At present, some of the Blast options we used are no longer available.

**Table 1:** Differences in Blastn hit numbers.

**Limit by Entrez Query**	**Descr#, Aligment#, Graph#**	**Hits#found**
1.-nothing	100, 100, 50	435
2.-nothing	5000, 5000, 1000	4903
3.-nothing	10000, 10000, 1000	4903
4.-Homo sapiens BUT NOT mitochondrion	100, 100, 50	116
5.-Homo sapiens BUT NOT mitochondrion	5000, 5000, 1000	2497

Data are obtained by comparing reference Human mt Genomes (J01415.2 in GenBank) with Human Nuclear DNA sequences in differing conditions. Maximum fixed Description number, Graphic display number and Alignments number do not fit Hits# obtained; thus, true hit number is that obtained when set values are higher than number of obtained hits.

**Table 2:** Differences in Blastn hit numbers by changing human genome searched datasets.

**Limit by Entrez query**	**Hits#**	**Type of selected data reported in the Blastn output**
6. – nothing	4903	Human Complete mt genomes, Human D-loop, other species
7. – Homo sapiens BUT NOT mitochondrion	2497	Genomic DNA, cDNA, D-loop also from other species
8. – Homo sapiens [ORGN]	4903	Human Complete mt genomes, D-loop,
9. – Homo sapiens [ORGN] NOT mitochondrion [PROP]	4903	Human Complete mt genomes, D-loop, other species
10. – Homo sapiens [ORGN] AND genomic DNA [MOLTYPE] NOT mitochondrion [PROP]	2154	Human Genomic DNA, cDNA, 2 complete mt genomes
11. – Homo sapiens [ORGN] NOT mitochondrion [ALL]	2497	Human Genomic DNA, cDNA, D-loop
12. – Homo sapiens [ORGN] AND genomic DNA [MOLTYPE] NOT mitochondrion [ALL]	123	Human Genomic DNA, cDNA
13. – Homo sapiens [ORGN] AND genomic DNA [MOLTYPE] NOT (mitochondrion OR mitochondrial) [ALL]	119	Human Genomic DNA
14. – nothing	16350	Genomic, D-loop, mt genomes other organisms
15. – Homo sapiens NOT mitochondrion	2097	Genomic DNA, D-loop, other organisms
16. – Homo sapiens [ORGN]	2106	Human Genomic DNA, D-loop, mt complete genomes
17. – Homo sapiens [ORGN] NOT (mitochondrion OR mitochondrial) [ALL]	2097	Human Genomic DNA, Human D-loop
18. – Homo sapiens [ORGN] NOT mitochondrion [PROP]	2154	2 human mt genomes, Genomic DNA
**19. – Homo sapiens [ORGN] NOT (mitochondrion OR mitochondrial) [ALL]**	**2145**	**HGPC+Celera+Assemblychr7**
20. – Homo sapiens [ORGN] NOT mitochondrion [ALL]	2145	HGPC+Celera

Different Hits number and different class of selected entries obtained by changing subject sequence datasets through "Limits by Entrez query" function. Resulting subject sequences are subsets of non-redundant nucleotide database (query 6 to 13), ref_seq genome database (query 14 to 17), Chromosome human genome database (query 18 to 20) all available through Blastn at NCBI. E-value set at 0.001. Description#, graphic display# and alignments# were set at maximum values allowed. Runs 19 and 20, resulting in 2145 hits, were those most suited to our needs, i.e., to select completely assembled human nuclear sequences.

### MegaBlast results

We used MegaBlast to compare the J01415.2 Human mt sequence against the last assembled Human genome database and Human Genome Reference sequence sets (Build 36.2, January 2007): when the E threshold was set at 0.001, the resulting hits were 288 and 186 respectively.

### BLAT results

We applied the BLAT program by submitting the J01415.2 Human mt sequence to the four available Human Builds, obtaining 118, 122, 124 and 117 hits for the hg15, hg16, hg17 and hg18 human genome assemblies. In parallel, we applied the same runs with the NC_001807.4 human mt genome, reported by NCBI as the reference human mt genome sequence. There are 22 differences between these two genomes, which cause differing results (data not shown). This is a further explanation of the differing results obtained in published compilations in which different query sequences were used.

### The RHNumtS compilation production

**Figure 2:**
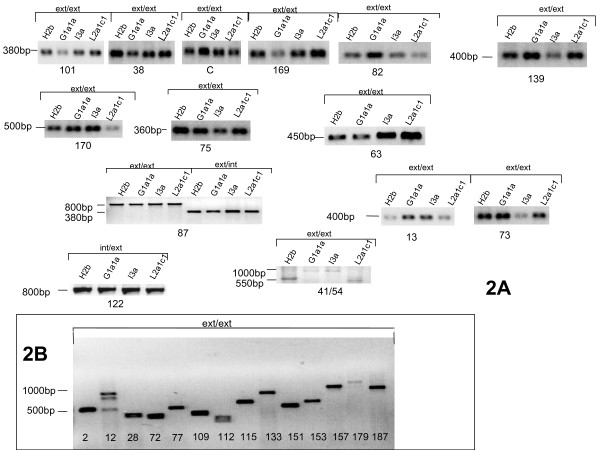
**PCR amplification of 41 selected NumtS**. PCR amplification of (a) 27 selected NumtS in 4 healthy subjects from different ethnic groups (haplogroups H2b (Europe), L2a1c1 (North Africa), I3a (Latin America) and G1a1a (Japan)); (b) 14 in the H2b sample only. Primers were designed with PRIMER3 software, testing the amplification of the full NumtS (external-external primers) or part of it (external-internal primers or internal-internal primers). In NumtS 41–54, samples H2b and L2a1c1 have shorter amplicons, due to a lower number of repetitions. Triple band in NUMTS 12 was due to aspecific amplification, subsequently reduced by increasing stringency of primers annealing. Abbreviations: ext for external, int for internal; the number below each band refers to the NumtS code assigned within the RHNumtS compilation.

Comparisons of our results with the published ones allowed us to produce the RHNumtS compilation. The resulting NumtS total 190. Each NumtS in the compilation refers to an mtDNA fragment; thus, if 2 mtDNA regions are contiguous on nuclear DNA, they are considered as two distinct NumtS. This is the case, for example, of the repeated NumtSs 41–54 that was here validated experimentally (see Figure 2a). Table 3 reports RHNumtS data for NumtS whose mt fragment length is greater than 2000 bp. The complete compilation is available in Additional file 1. The rationale used to produce the compilation is described here. The reference results are those obtained through BlastN (code 19 in Table 2). Hits less than 2000 nucleotides from each other, on both nuclear and mt genomes were merged. The results were compared with those from Megablast [Human Genome all assemblies (Build 36.2, January 2007)] and BLAT (Assembling hg18): the more reproducible the results obtained with the various methods, the higher the probability that NumtS exists in the Human Genome. Each NumtS in RHNumtS is identified by a numeric code (RHNumtS identifier); only three were identified by a letter. To each NumtS we associated: chromosome and strand location, both mt and nuclear coordinates of the NumtS ("mt start" and "mt end", "chr start" and "chr end"); mitochondrial and nuclear fragment lengths and differences between mitochondrial and nuclear fragment lengths; the longer the NumtS, the higher the number of gaps within the NumtS, thus indicating that it likely underwent several modifications since the time of its insertion in the nuclear genome. Additional file 2 reports the comparison of Blastn obtained data vs BLAT and Megablast results. Of the 190 NumtS available through RHNumtS, 122 (64%) were matched with both Blastn and BLAT, 60 (32%) with Blastn only and eight (4%) with Blastn, but located on the Celera assembly instead of the public Human Genome consortium. The compilation was compared with some published compilations. For each NumtS, Additional file 3 lists its presence (OK) or absence (-) in some of the published compilations. Question marks (?) indicate ambiguous cases. With respect to the Parr compilation [32], the sequenced NumtS are all present in RHNumtS; this validates our results, although some sequenced NumtS could not be present in our compilation, due to their polymorphic features. The same applies to the two NumtS sequenced by Collura [37], located on chromosome 7. In order to quantify the strength of our approach, RHNumtS quality scores were assigned to each NumtS for each program applied and for each match with the selected published compilations, according to defined criteria: 0.25 or 0.50 for an ambiguous or perfect match with each of the selected published compilations [16,23,26,28], 2.00 for matches with Parr and Collura sequenced NumtS, 0.25 for ambiguous matches with Megablast hits, 1.00 for perfect matches with MegaBlast and BLAT hits, and 0.75 for NumtS not directly identified by BLAT. Score values range from 6 to 0.25. The last column of Additional file 1 and Table 3 lists the total score for each NumtS. NumtS with scores higher than 3 (16.3% of the total Reference Human NumtS compilation) are highlighted. Perfect matches with published compilations are at most 12%: this is the case of the comparison with the Wallace compilation [26].

**Table 3:** Longest NumtS of Reference Human NumtS compilation (RHNumtS)

**NumtS Code**	**Chr**	**Strand**	**Mt Start**	**Mt End**	**Mt fragment length**	**Nuc Start**	**Nuc End**	**Chr fragment length**	**Difference**	**Quality Score**
1	1	+	3914	9755	5842	554327	560167	5840	3	4
3	1	-	6060	9316	3257	107146786	107150029	3243	15	1.25
4	1	-	1051	3162	2112	120286496	120288780	2284	173	0.75
9	1	-	9782	13593	3812	233768514	233772288	3774	39	1.75
10	1	-	636	6189	5554	236170699	236176250	5551	4	1.75
11	1	-	12218	16563	4346	236177249	236181582	4333	14	1.75
14	2	+	12220	16475	4256	82896241	82900506	4265	10	1.75
19	2	+	596	5892	5297	117495259	117500547	5288	10	3.25
20	2	+	9196	13574	4379	120685762	120690928	5166	788	1.75
22	2	+	3799	15354	11556	130745853	130757329	11476	81	2
23	2	-	10657	15398	4742	131843104	131847799	4695	48	1.75
24	2	-	3799	10519	6721	131853669	131860205	6536	186	1.5
25	2	-	598	5892	5295	140691291	140698242	6951	1657	3.5
27	2	-	9166	16563	7398	143566386	143574013	7627	230	1.75
30	2	+	11801	15067	3267	155875844	155879111	3267	1	2
33	2	-	10440	13131	2692	201785264	201787949	2685	8	2
34	2	+	6966	11240	4275	203187200	203191742	4542	268	2
36	2	-	596	3105	2510	212346765	212349578	2813	304	1.75
37	2	+	4854	7590	2737	212350179	212352885	2706	32	1.5
58	3	-	6604	9316	2713	89718693	89721366	2673	41	2
61	3	-	9787	12340	2554	108095676	108098627	2951	398	1.25
62	3	+	13536	15573	2038	108100533	108101514	981	1058	1.5
71	4	-	9781	12301	2521	25328634	25331437	2803	283	1.75
76	4	-	9485	16561	7077	65155336	65160181	4845	2233	1.75
79	4	+	596	3105	2510	117438367	117440855	2488	23	1.75
81	4	-	672	15325	14654	156592474	156607061	14587	68	3.5
88	5	-	341	2697	2357	79981597	79983943	2346	12	4.75
89	5	+	12662	16124	3463	93928917	93932379	3462	2	5
91	5	-	6117	15183	9067	99409541	99418648	9107	41	4.5
94	5	-	10270	15488	5219	134286898	134292116	5218	2	5
97	6	+	8437	10622	2186	92493159	92493750	591	1596	1.5
100	6	+	7451	11649	4199	154028400	154032608	4208	10	1.75
102	7	+	8505	15238	6734	57238827	57245471	6644	91	3.75
103	7	+	3819	15924	12106	57257414	57269467	12053	54	6
104	7	-	3117	11880	8764	63201998	63210482	8484	281	1.5
105	7	-	5513	8246	2734	68433640	68436926	3286	553	1.75
106	7	+	13065	15369	2305	111799937	111802234	2297	9	2
107	7	-	2793	6553	3761	141147677	141151744	4067	307	2
108	7	+	600	3095	2496	142052596	142055088	2492	5	2.25
110	8	+	636	4888	4253	32988565	32992739	4174	80	1.5
113	8	-	656	4880	4225	47858273	47861837	3564	662	4
114	8	-	9176	16569	7394	68655653	68662552	6899	496	1.75
117	8	+	1013	7114	6102	104164459	104171823	7364	1263	3.5
120	9	+	1294	13574	12281	5082095	5100699	18604	6324	1.5
121	9	+	598	3093	2496	33646633	33649128	2495	2	1.5
125	9	-	4773	6873	2101	82368550	82370501	1951	151	1.75
126	9	+	9202	11598	2397	93911111	93913772	2661	265	3.25
128	10	+	2417	4831	2415	20075681	20077114	1433	983	2.25
131	10	+	636	3105	2470	57027643	57030440	2797	328	1.5
132	10	-	3821	7698	3878	71020912	71025687	4775	898	1.75
134	11	-	577	2972	2396	10486010	10488403	2393	4	6
140	11	+	9820	15243	5424	80940264	80945683	5419	6	1.75
142	11	-	724	9666	8943	102778067	102786933	8866	78	3.25
150	13	+	13052	16472	3421	95142796	95146598	3802	382	1.5
156	14	+	11367	15325	3959	83708940	83713093	4153	195	1.75
158	15	+	9786	15318	5533	56229853	56235023	5170	364	1.5
159	16	-	2468	7683	5216	3357487	3362068	4581	636	3.5
160	16	-	8688	15327	6640	10720543	10726494	5951	690	1.5
164	17	-	596	5979	5384	19442485	19449425	6940	1557	3.5
165	17	+	14365	16569	2205	21942648	21944853	2205	1	3.75
166	17	+	1	11112	11112	21944854	21955968	11114	3	3.5
171	20	-	649	4038	3390	55366111	55369449	3338	53	3.5
174	X	-	581	5892	5312	55221910	55227180	5270	43	4
175	X	+	1049	3161	2113	61976282	61978565	2283	171	1.75
182	X	+	1054	4415	3362	142345841	142349570	3729	368	1.75
184	Y	+	596	4477	3882	8294669	8300289	5620	1739	1.25

Each NumtS was assigned an identifying numeric code, according to increasing values starting from chromosome 1; a letter code (A, B, or C) was assigned to only 3 NumtS, because they were located later, when all other NumtS had already been characterised. Chromosome and strand location is listed for each NumtS; both mt and nuclear coordinates of NumtS ("mt start" and "mt end", "chr start" and "chr end"); mitochondrial and nuclear fragment lengths; "difference" between mitochondrial and nuclear fragment lengths; and RHNumtS quality score are also reported. Additional file 1 reports the complete RHNumtS compilation: there, NumtS exclusively identified by Blastn are shown in grey and NumtS exclusively identified by Blastn, but only on Human Genome Celera Assembly, shown in black in columns "Nuc start" and "Nuc end"; repeated NumtS in bold type and underlined in columns "Mt start" and "Mt end". NumtS with scores higher than 3 are shown in grey in column "score".

### Human NumtS Sequencing

The assigned scores are theoretical indicators of the quality of our prediction, but experimental validation of the predicted RHNumtS compilation is definitely a must and this is our goal in the immediate future. Starting from the RHNumtS compilation, we propose to test the real presence of each NumtS in a set of different healthy subjects belonging to different geographic areas and various haplogroups, in order to verify if the NUMTS presence/absence may be different in various phylogenetic lineages. Experimental validation will be based on the amplification and sequencing of the NumtS. This requires a great effort in terms of manpower and funds. However such project is currently ongoing and here we report only preliminary results obtained for 41 of the 190 NumtS, selected among the NumtS with lower scores. Indeed, the lower the score, the higher the probability that the NumtS is a false positive. Table 4 reports the list of the analyzed NumtS with information about samples where amplification and sequencing has been successful. Figure 2 reports the PCR amplification of (a) 27 of the selected NumtS in 4 healthy subjects from different ethnic groups and (b) 14 NumtS in a European sample. With respect to NumtS 41–54, the H2b and L2a1c1 samples have shorter amplicons, due to the presence of a lower number of repeats, as confirmed in the multi-alignment (Additional file 4).

**Table 4:** Amplified and sequenced NumtS

**NumtS Code**	**Amplified Haplogroup samples**	**Sequenced Haplogroup samples**
2	H2B	H2B
12	H2B	H2B
13	H2B, L2a1c1,G1a1a, I3a	H2B
28	H2B	H2B
38	H2B, L2a1c1,G1a1a, I3a	H2B
41	H2B, L2a1c1,G1a1a, I3a	H2B, L2a1c1,G1a1a, I3a
42	H2B, L2a1c1,G1a1a, I3a	H2B, L2a1c1,G1a1a, I3a
43	H2B, L2a1c1,G1a1a, I3a	H2B, L2a1c1,G1a1a, I3a
44	H2B, L2a1c1,G1a1a, I3a	H2B, L2a1c1,G1a1a, I3a
45	H2B, L2a1c1,G1a1a, I3a	H2B, L2a1c1,G1a1a, I3a
46	H2B, L2a1c1,G1a1a, I3a	H2B, L2a1c1,G1a1a, I3a
47	H2B, L2a1c1,G1a1a, I3a	H2B, L2a1c1,G1a1a, I3a
48	H2B, L2a1c1,G1a1a, I3a	H2B, L2a1c1,G1a1a, I3a
49	H2B, L2a1c1,G1a1a, I3a	H2B, L2a1c1,G1a1a, I3a
50	H2B, L2a1c1,G1a1a, I3a	H2B, L2a1c1,G1a1a, I3a
51	H2B, L2a1c1,G1a1a, I3a	H2B, L2a1c1,G1a1a, I3a
52	H2B, L2a1c1,G1a1a, I3a	H2B, L2a1c1,G1a1a, I3a
53	H2B, L2a1c1,G1a1a, I3a	H2B, L2a1c1,G1a1a, I3a
54	H2B, L2a1c1,G1a1a, I3a	H2B, L2a1c1,G1a1a, I3a
63	H2B, L2a1c1,G1a1a, I3a	H2b
72	H2B	H2B
73	H2B, L2a1c1,G1a1a, I3a	H2B
75	H2B, L2a1c1,G1a1a, I3a	H2B
77	H2B	H2B
82	H2B, L2a1c1,G1a1a, I3a	H2B
87	H2B, L2a1c1,G1a1a, I3a	H2B, L2a1c1,G1a1a, I3a
101	H2B, L2a1c1,G1a1a, I3a	H2B
109	H2B	H2B
112	H2B	H2B
115	H2B	H2B
122	H2B, L2a1c1,G1a1a, I3a	H2B, L2a1c1,G1a1a
133	H2B	H2B
139	H2B, L2a1c1,G1a1a, I3a	H2B
151	H2B	H2B
153	H2B	H2B
157	H2B	H2B
169	H2B, L2a1c1,G1a1a, I3a	H2B
170	H2B, L2a1c1,G1a1a, I3a	sequencing failed
179	H2B	sequencing failed
187	H2B	sequencing failed
C	H2B, L2a1c1,G1a1a, I3a	H2B

For each of the 41 analysed NumtS, the mt haplogroup code of the sample, if amplified and if sequenced, is reported. NumtS 170, 179 and 187 in the H2b sample and 122 in the I3a sample have not been sequenced because primers were not optimal for sequencing.

The 122 NumtS sequence of the Latin American sample and the European sample of NumtS 170, 178 and 187 were not obtained. For NumtS 87, 122 and 41–54 Additional file 4 reports the nucleotide multi-alignment of the amplified and sequenced NumtS in the samples from the 4 different haplogroups, compared with the NumtS sequence as extracted from the Human Genome build 36.2 through the UCSC genome browser (hg18 release), and the sequences of the corresponding mitochondrial region for the same samples. As it appears from the multi-alignment there is a high conservation of the NumtS among the different subjects, although heterozygous sites can be observed (nucleotide ambiguity letter such as Y for C/T, R for A/G, etc.). NumtS sites reporting ambiguous nucleotides do in fact refer to sites where two alleles are evidenced in the sequence. The comparison of NumtS sequence with the corresponding mtDNA also clearly shows divergence among them. Additional file 5 reports the multialignments of the other 21 NumtS whose sequences have been so far produced for the European sample only. The NumtS sequence is aligned with the hg18 and mitochondrial corresponding sequences.

### NumtS features

The resulting compilation was further analyzed, in order to qualify and quantify the process of transfer of Human mtDNA into the nuclear genome.

#### NumtS distribution along the genome

Chromosome 2 hosts the largest amount of NumtS, whereas chromosomes 19 and 22 do not show to harbour NumtS although, due to their polymorphic features, some individuals may present them on the latter chromosomes. Generally speaking, the longer the chromosome, the greater the chance of locating NumtS, partly because, as reported below, selection operates in the direction of avoiding NumtS inside genes, so that shorter chromosomes with a higher density of genes are less prone to hosting NumtS. In addition, with respect to the chromosome region where NumtS are located, no preference between euchromatin or etherochromatin regions has been observed. However, for each NumtS chromosome band, Additional file 6 lists information contributing to an overview about where NumtS are integrated.

#### NumtS dimensions

For each NumtS Additional file 1 also lists the mt and chromosomal fragment lengths, besides the mt and nuclear coordinates. These values do not coincide since, after insertion, NumtS undergo rearrangements such as deletions, insertions, and single nucleotide substitutions. The older the NumtS, the greater the difference between mitochondrial and chromosomal fragments (see difference column in Table 3 and additional file 1). The longest mt fragment located on the nuclear genome is NumtS no. 81, 14654 mt bp, located on chromosome 4 and highly compacted: the 81 NumtS chromosomal fragment is in fact 14587 bp long. The shortest NumtS is 45 bp long. More than 30% of NumtS derive from mt fragments longer than 2000 mt bp (Table 3). NumtS chromosomal fragment length is listed in Additional File 6: the longest one is NumtS 120, 18604 chromosomal BP, containing a mt fragment 12281 bp long, located on chromosome 9, with an RHNumtS score of 1.5. NumtS 103, 12053 bp long and derived from an mt fragment of 12106 nucleotides, located on chromosome 7, received a score of 6, because it was entirely sequenced from both Parr [32] and Collura [35].

#### Estimation of similarity

Although Blastn, Megablast and BLAT provide scores and percentages of identity, the values are approximate for each hit, due to the heuristic algorithms implemented in these programs. We thus further analysed each NumtS by applying both the Needleman and Wunsch algorithm for global alignment and the Waterman and Smith algorithm for local ones. Additional file 7 shows alignment scores compared with BLAT scores. The highest score is that of NumtS 81, which contains the longest mt fragment. Half of the NumtS have similarity values between 99% and 80%, thus showing a high degree of conservation from the time of their insertion in the nucleus.

#### NumtS in nuclear genes

Once the NumtS had been located through the UCSC Genome Browser [38] and NCBI Map viewer [39], we checked their location in nuclear genes. For NumtS located in genomic regions coding for genes, Additional file 6 lists both the gene name and the region of the gene where the NumtS are mapped. There are 16 NumtS inside genes; they are always located inside introns, and only two (13 and 88) are located in 5'UTR regions.

#### NumtS and isochores

Isochores are large DNA segments (> 300 kb on average) characterized by an internal variation in GC well below the full variation observed in the mammalian genome [40]. The previous definition of human isochores, based on ultracentrifugation in Cs_2_SO_4 _density gradients, has recently been revised by simply scanning the GC% content along the entire genome, and the five isochore families L1, L2, H1, H2 and H3, were defined according to GC content, values increasing from L1 to H3. We mapped the NumtS on isochores according to the data published in [41]. Results are listed in Additional file 6. Only 9% of total NumtS in RHNumtS were not located within isochores; 5% maps with the highest GC dense isochores (H2, H3); 30% on isochore H1, and 33% and 23% on isochores L2 and L1, respectively. Thus, NumtS prefer locations with low GC contents, corresponding to poor gene-containing regions. Indeed, the presence of a NumtS inside a gene may cause loss of function, so selection may act to clean out the genome from disrupting NumtS insertion events. This is also confirmed by the fact that, when NumtS are located in a gene, the gene region is always an intron and in some rare cases a UTR.

#### Mapping of Human NumtS along Human mt genome

**Figure 3:**
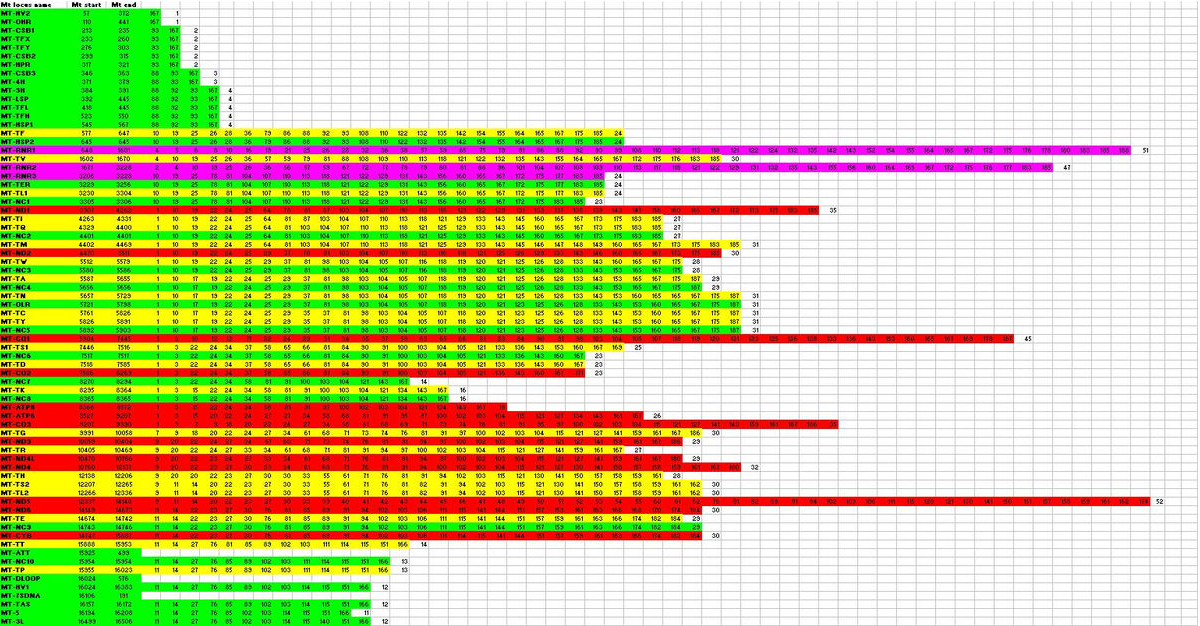
**Mapping of Human NumtS on Human mitochondrial genome**. Column 1 reports mt gene name, column 2 and 3 report location of the mt gene inside the genome and, starting from column 4, the RHNumtS identifiers of the NumtS containing the mt gene are shown. An RHNumtS identifier present in three contiguous genes indicates that NumtS contains the centrally located gene in its entire length; otherwise, it is partially contained. Green: regulatory regions; yellow: tRNA genes; red: protein coding genes; pink: rRNA genes.

As already reported by Parr et al. [32], human NumtS are made up of mt fragments covering the entire human mt genome, "the pseudo-mitochondrial human genome". For each human mt gene, Figure 3 shows the RHNumtS identifiers containing it: all mt regions are present in the NumtS, but the number of NumtS containing any mt gene is highly variable. Moreover, not all NumtS contain an mt gene entirely, because the locus may be located partially at the 5' and 3' ends of the NumtS or because it may have been truncated after insertion by rearrangement events. The mt genes most frequently present in the "pseudo-human mt-genome" are the two ribosomal RNAs, ND5 and COI genes; the least represented is the D-loop region. At present, no explanation for such preferences can be made. Certainly, the higher the number of locus copies in the nuclear genome, the higher the risk of co-amplification of mt-nuclear DNA, in any study on mtDNA variations.

## Conclusions

The RHNumtS compilation proposed here results from the application of several bioinformatics approaches and from comparisons of resulting data with previously published Human NumtS compilations. It thus represents a highly reliable reference basis on which to start designing a lab protocol to test the truthfulness of each NumtS. Two experimental procedures are proposed: nDNA-mtDNA hybridisation, as already done with the *Canis familiaris *genome [42], or by amplifying and sequencing NumtS themselves. The latter approach, here adopted to validate 25% of the NumtS whose score in our compilation is lower than 3, confirms the quality of our bioinformatics approach; however, a systematic and complete experimental validation will be designed. In parallel, we are already designing the RHNumtS database structure for implementation in the HmtDB resource [43]. This database will report the NumtS sequences annotated with the attributes derived from both *in silico *and *in vitro *analyses. This work will be important because until now no database concerning NumtS has ever been published, so that we will be able to offer an updated reference for consultation on-line to the scientific community interested in analysis of foreign DNA integration. In the future, the same database will host NumtS compilations from other organisms, but these will be generated only when the nuclear genome of a specific organism has reached a high-quality level of assembly. Once the Reference Compilations for other organisms have been produced, pairwise NumtS compilation comparisons, based on the UCSC Genome Browser Alignment net option, will be used to define orthologous NumtS. This procedure has been already implemented in [31] in the Human-Chimp comparison. Lastly, some features of Human NumtS will be investigated. We will also analyze SNPs located inside NumtS through dbSNP at NCBI. These data may offer new perspectives in population mitochondrial genetics, preferentially in those NumtS that comparative genomics may indicate as being conserved. Lastly, with respect to the NumtS insertion site, we have started some preliminary pattern analysis by applying the WORDUP program [44] to the NumtS flanking region, although no significant results have yet been found (data not shown). This should mean that NumtS integration is not guided by specific DNA signals or does not generate any specific pattern, as is the case for retrotransposons, in which LTR sequences are generated after integration. Gherman et al. have recently confirmed the randomness of NumtS site integration [12].

## Methods

### Blast

Blastn compares a nucleotide query sequence against a nucleotide database. It can produce differing results if the parameters selected among those available differ. The input data for a Blastn run are: query sequence, sequence set to be searched, Expected number of chance matches in a random model (E-value), maximum number of hits to be displayed, maximum number of aligned sequences to be displayed, size of the string to be searched in pairwise comparison (word size), scoring parameters, and filtering and masking options. In addition, within the "sequence set to be searched" section, a specific sequence subset can be selected with the Limits option, available through the *Entrez *retrieval system [45]. We submitted several runs, changing: a) the sequence set to be searched ("chromosome", "nr" (not redundant nucleotide sequences) or "refseq_genome"), with and without Limits by *Entrez*; b) Maximum number of Hits to be displayed (egg. Description# = 1000, Graphic display# = 500, Alignments view# = 1000). The threshold E-value was always fixed at 0.001. The query sequence was that of the revised Reference Cambridge Sequence (GenBank Accession J01415.2, [14]). As already stated above, if the reference human mitochondrial genome is changed, the Blast result also changes.

### MegaBlast

As reported in the NCBI Handbook [36], "MEGABLAST is specifically designed to efficiently find long alignments between very similar sequences and thus it is the best tool to find the identical match to the query sequence. In addition to the expected value significance cut-off, MEGABLAST also provides an adjustable percent identity cut-off that overrides the significance threshold."

### BLAT

BLAT (BLAST-Like Alignment Tool) [37] is a very fast sequence alignment tool similar to BLAST. On DNA queries, BLAT is designed to find quickly sequences with 95% or greater similarity 40 BP long or more. It may miss genomic alignments that are more divergent or shorter than these minima, although it will find perfect sequence matches of 33 bases and sometimes as few as 22. The tool is capable of aligning sequences containing large intron sequences. Thus, because NumtS, after insertion in the nuclear genome, undergoes further arrangements, losing or acquiring new interspersed fragments and/or single nucleotides, BLAT is definitely a good tool for locating them. Instead, Blast locates single fragments of the entire NumtS. As both approaches are useful for a complete view of NumtS, this is the reason for our using both methods. The BLAT program available at the UCSC site has the great advantage of allowing the comparison of a query sequence against a repertoire of four different Human Builds, starting from April 2004 up to October 2006. Each Human Build also reports the absolute coordinate for each Chromosome, thus ensuring a good referencing system for locating NumtS in the genome. Each hit in BLAT corresponds to a wide region where several blocks are located. These can be displayed and analysed starting from the BLAT output page and clicking on "details", so that the sequences of each block and their alignment appear. Thus, aligned blocks with gaps less than or equal to 8 BP are merged, when only one sequence has a gap or when gaps in both sequences are of the same size. This implies that the identity percentage is the sum of the matches in each block divided by the sum of the block lengths.

### Amplification and Sequencing

In order to carry out a preliminary validation of the compilation, we selected 41 NumtS whose score is lower than 3 and submitted them to PCR amplification and sequencing. The 41 NumtS were amplified on DNA extracted from the blood of a European individual available in G. Romeo laboratory. Moreover 27 among the 41 NumtS were also amplified from DNA extracted from the blood of 3 healthy subjects belonging to different geographic areas and different haplogroups, in order to verify the NumtS presence/absence in phylogenetic lineages. Samples selected for analysis were individuals coming from Japan (A. Torroni laboratory), Latin America and North Africa (V. Carelli laboratory), and belonged respectively to haplogroups H2b, G1a1a, I3a and L2a1c1. Among the 27 NumtS, 16 were sequenced in all the samples: NumtS 87, 122 and 41–54. NumtSs 41–54, because they were tandemly repeated, were amplified and sequenced all together. For some of the amplified NumtS, the sequencing failed. These are marked in Table 4. PCR conditions were not always equal. Primers were designed with PRIMER3 software, testing the amplification of the full NumtS (external-external primers) or part of it (external-internal primers or internal-internal primers) as reported in Table 4 and Figures 2a and 2b. Before the application of the PRIMER3 program to the NumtS sequence and its flanking regions, the sequence was submitted to further bioinformatics test by blasting it against the J01415.2 reference mt sequence using the Blast2 program [49].

This produces new results allowing a refinement of the RHNumtS compilation as far as it concerns NumtS region extension.

Primer sequences are available on request. The sequences were produced starting from the amplified fragments. Sequencing was performed with BigDye v3.1 (Applied Biosystems, Foster City, CA), according to the manufacturer's instructions, on an AB3730 capillary analyzer. The produced sequences have been multialigned by applying MAFFT [46] and MUSCLE [47] programs both available at [48] in the tools section.

## Authors' contributions

MA: study design and coordination; SC and DL: bioinformatics analysis and results organization; GG: NumtS amplification and sequencing; CS: supervisor; GR: supervisor and sequencing fund support. All authors discussed the data and participated in the production of the manuscript.

## Supplementary Material

Additional file 1 **Complete reference Human NumtS compilation (RHNumtS)**. A detailed legend is reported in Table 3. Click here for file

Additional file 2 **Comparison of BLAT and Megablast results**. For each NumtS in the RHNumtS compilation, Megablast and BLAT programs either did or did not detect it (yes/no); if yes, table shows mt coordinates, BLAT code assigned, number of aligned blocks and fragments as reported in BLAT details, and %identity value as reported in BLAT output. NumtS reporting code "Yes_1" in "BLAT hits correspondence table" were recovered by submitting mt fragment corresponding to a NumtS obtained through Blastn to BLAT, and not through submission of entire mt genome (J01415.2 or NC_01807.4) to BLAT. Click here for file

Additional file 3 **Comparison between Reference Human NumtS compilation and some published compilations**. Columns 2–7 list reference numbers, as reported in reference section. "-": absence of NumtS in corresponding reference, "OK": perfect match, "?" : partial match. Click here for file

Additional file 4 **Multi-alignments of sequenced NumtS from 4 different phylo-geographic samples.** The nucleotide multi-alignments of the amplified (fig 2a in the manuscript) and sequenced NumtS 87, 122 and 41–54 of individuals coming from Europe, Japan, Latin America and North Africa belonging respectively to haplogroups H2b, G1a1a, I3a and L2a1c1 compared with the NumtS sequence as it can be extracted from the Human Genome build36.2 through the UCSC genome browser (hg18 release) and the sequences of the corresponding mitochondrial region for the same samples, are reported. Multi-alignments of NumtS 87 and 122 include the rCRS sequence (accession number J01415.2 in GenBank) also, thus allowing the exact localization of the variant sites respect to the universally used human mitochondrial reference sequence. As far as NumtS 41-54 the reference sequence has been added to NumtS 43 only in a distinctly reported multialignment.  The conservation of NumtS is evident from the multialignment among the different subjects, although heterozygous sites can be observed (nucleotide ambiguity letter such as Y for C/T, R for A/G etc.). Each multi-alignment refers to the nuclear region (from Chromosome start to Chromosome end), as reported in additional file 1. The NumtS sequences produced in our validation experiments are named with a code defined by the NumtS code and the haplogroup of the sample. The corresponding mtDNA regions are coded as mt, followed by the haplogroup code. The reference sequence for 122 NumtS is extracted from Celera genome chromosome 9 (CM00260). Sequencing of L2a1c1 122 NumtS, H2b and L2a1c1 41, 42 and 54 NumtS failed. Click here for file

Additional file 5 **Sequences of 22 NumtS from a European sample (haplogroup L2a1c1).** Each sequence has been multi-aligned with the NumtS sequence as it can be extracted from the Human Genome build36.2 through the UCSC genome browser (hg18 release), the sequence of the corresponding mitochondrial region for the same sample and the rCRS sequence (accession number J01415.2 in GenBank ). Each multi-alignment refers to the nuclear region (from Chromosome start to Chromosome end), as reported in additional file 1. The NumtS sequences produced in our validation experiments are named with a code defined by the NumtS code and the haplogroup of the sample. The corresponding mtDNA regions are coded as mt, followed by the haplogroup code. Click here for file

Additional file 6 **NumtS in RHNumtS featuring data**. Scores obtained through application of NeedleN program. Chromosome map location, isochore family in which NumtS is located, name of gene and its region, if any, and NumtS location are all listed. NumtS are ordered according to decreasing length. Click here for file

Additional file 7 **Alignment results obtained with NeedlN and WaterN programs, available through EBI SRS server, compared with BLAT scores**. Table shows results for each NumtS from both Needleman and Wunsch and Waterman and Smith algorithms for global and local alignments. Results of alignments are compared with BLAT scores. Each NumtS is identified by a numeric code in NumtS Code column. Columns list % similarity, % gaps, score, alignment length and ratio obtained by application of alignment programs. BLAT scores for each NumtS Code are shown in last column. Click here for file

## References

[B1] Bensasson D, Zhang D, Hartl DL, Hewitt GM (2001). Mitochondrial pseudogenes: evolution's misplaced witnesses. Trends Ecol Evol.

[B2] Du Buy HG, Riley FL (1967). Hybridization between the nuclear and kinetoplast DNAs of *Leishmania enriettii *and between nuclear and mitochondrial DNAs of mouse liver. Proc Nat Acad Sci USA.

[B3] Farrelly F, Butow RA (1983). Rearranged mitochondrial genes in the yeast nuclear genome. Nature.

[B4] Gellissen G, Bradfield JY, White BN, Wyatt GR (1983). Mitochondrial DNA sequences in the nuclear genome of a locust. Nature.

[B5] Wright RM, Cummings DJ (1983). Integration of mitochondrial gene sequences within the nuclear genome during senescence in a fungus. Nature.

[B6] Jacobs HT, Posakony JW, Grula JW, Roberts JW, Xin JH, Britten RJ, Davidson EH (1983). Mitochondrial DNA sequences in the nuclear genome of *Strongylocentrotus purpuratus*. J Mol Biol.

[B7] Tsuzuki T, Nomiyama H, Setoyama C, Maeda S, Shimada K (1983). Presence of mitochondrial DNA like sequences in the human nuclear DNA. Gene.

[B8] Kemble RJ, Mans RJ, Gabay-Laughnan S, Laughnan JR (1983). Sequences homologous to episomal mitochondrial DNAs in the maize nuclear genome. Nature.

[B9] Hadler HI, Dimitrijevic B, Mahalingam R (1983). Mitochondrial DNA and nuclear DNA from normal rat liver have a common sequence. Genomics.

[B10] Lopez JV, Yuhki N, Masuda R, Modi W, O'brien SJ (1994). *Numt *a recent transfer and tandem amplification of mitochondrial DNA to the nuclear genome of the domestic cat. J Mol Evol.

[B11] Bravi CM, Parson W, Bandelt V, Macaulay M, Richards HJ (2006). Numts Revisited. Human Mitochondrial DNA and the Evolution of Homo Sapiens.

[B12] Gherman A, Chen PE, Teslovich TM, Stankiewicz P, Withers M, Kashuk CS, Chakravarti A, Lupski JR, Cutler DJ, Katsanis N (2007). Population Bottlenecks as a Potential Major Shaping Force of Human Genome Architecture. PLoS Genet.

[B13] Zischler H, von Haeseler A, Paabo S (1995). A nuclear fossil of the mitochondrial D-loop and the origin of the modern humans. Nature.

[B14] Andrews RM, Kubacka I, Chinnery PF, Lightowlers RN, Turnbull DM, Howell N (1999). Reanalysis and revision of the Cambridge reference sequence for human mitochondrial DNA. Nat Genet.

[B15] Yuan JD, Shi JX, Meng GX, An LG, Hu GX (1999). Nuclear pseudogenes of mitochondrial DNA as a variable part of the human genome. Cell Res.

[B16] Ricchetti M, Tekaia F, Dujon B (2004). Continued colonization of the Human Genome by mitochondrial DNA. Plos Biology.

[B17] Antunes A, Ramos MJ (2005). Discovery of a large number of previously unrecognised mitochondrial pseudogenes in fish genomes. Genomics.

[B18] Venkatesh B, Dandona N, Brenner S (2006). Fugu genome does not contain mitochondrial pseudogenes. Genomics.

[B19] Behura SK (2007). Analysis of nuclear copies of mitochondrial sequences in honey bee Apis mellifera genome. Mol Biol and Evol.

[B20] Woodward SR, Weyand NJ, Bunnell M (1995). DNA sequence from Cretaceous period bone fragments. Science.

[B21] Wallace DC, Stugard C, Murdock D, Schurr T, Brown MD (1997). Ancient mtDNA sequences in the human nuclear genome: a potential source of errors in identifying pathogenic mutations. Proc Natl Acad Sci USA.

[B22] Adcock GJ, Dennis ES, Easteal S, Huttley GA, Jermiin LS, Peacock WJ, Thorne A (2001). Mitochondrial DNA sequences in ancient Australians: Implications for modern human origins. Proc Natl Acad Sci USA.

[B23] Hazkani-Covo E, Sorek R, Graur D (2003). Evolutionary dynamics of large NUMTs in the Human Genome: rarity of independent insertions and Abundance of Post-insertion duplications. J Mol Evol.

[B24] Schmitz J, Piskurek O, Zischler H (2005). Forty million years of independent evolution: a mitochondrial gene and its corresponding nuclear pseudogene in primates. J Mol Evol.

[B25] Lopez JV, Culver M, Stephens JC, Johnson WE, O'Brien SJ (1997). Rates of nuclear and cytoplasmic mitochondrial DNA sequence divergence in mammals. Mol Biol Evol.

[B26] Mishmar D, Ruiz-Pesini E, Brandon M, Wallace DC (2004). Mitochondrial DNA like sequences in the Nucleus (NUMTs):Insights onto our african origins and the mechanism of foreign DNA integration. Human mutation.

[B27] Tourmen Y, Baris O, Dessen P, Jacques C, Malthiery Y, Reynier P (2002). Structure and chromosomal distribution of human mitochondrial pseudogenes. Genomics.

[B28] Mourier T, Hansen AJ, Willerslev E, Arctander P (2001). The Human Genome Project reveals a continuous transfer of Large Mitochondrial Fragments to the Nucleus. Mol Biol and Evol.

[B29] Woischnick M, Moraes CT (2002). Pattern of organization of human mitochondrial pseudogenes in the nuclear genome. Genome Res.

[B30] Bensasson D, Feldman MW, Petrov DA (2004). Rates of DNA duplication and Insertion in the Human Genome. J Mol Evol.

[B31] Hazkani-Covo E, Graur D (2007). A Comparative Analysis of Numt Evolution in Human and Chimpazee. Mol Biol Evol.

[B32] Parr RL, Maki J, Reguly B, Dakubo GD, Aguirre A, Wittock R, Robinson K, Jakupciak JP, Thayer RE (2006). The pseudomitochondrial genome influences mistakes in heteroplasmy interpretation. BMC Genomics.

[B33] Altschul SF, Gish W, Miller W, Myers EW, Lipman DJ (1990). Basic local alignment search tool. J Mol Biol.

[B34] Altschul SF, Boguski MS, Gish W, Wootton JC (1994). Issues in searching molecular sequence databases. Nature Genet.

[B35] Kent WJ (2002). BLAT – The BLAST-Like Alignment Tool. Genome Res.

[B36] Madden T The BLAST sequence analysis tool. NCBI Handbook part 3.

[B37] Collura R, Stewart CB (1995). Insertions and duplications of mtDNA in the nuclear genomes of Old World Monkeys and Hominoids. Nature.

[B38] UCSC Genome Browser. http://genome.ucsc.edu/.

[B39] NCBI Map viewer. http://www.ncbi.nlm.nih.gov/mapview.

[B40] Bernardi G (2000). Isochores and the evolutionary genomics of vertebrates. Gene.

[B41] Costantini M, Clay O, Auletta F, Bernardi G (2006). An isochore map of human chromosomes. Genome Res.

[B42] Ishiguro N, Nakajima A, Horiuchi M, Shinagawa M (2002). Multiple nuclear pseudogenes of mitochondrial DNA exist in the canine genome. Mamm Genome.

[B43] Attimonelli M, Accetturo M, Santamaria M, Lascaro D, Scioscia G, Pappada G, Russo L, Zanchetta L, Tommaseo-Ponzetta M (2005). HmtDB, a Human Mitochondrial Genomic Resource Based on Variability Studies Supporting Population Genetics and Biomedical Research. BMC Bioinformatics.

[B44] Pesole G, Prunella N, Liuni S, Attimonelli M, Saccone C (1992). WordUP: an efficient algorithm for discovering statistically significant patterns in DNA sequences. Nucl Acids Res.

[B45] Entrez home. http://www.ncbi.nlm.nih.gov/Entrez.

[B46] Katoh K, Misawa1 K, Kuma K, Miyata T (2002). MAFFT: a novel method for rapid multiple sequence alignment based on fast Fourier transform. Nucleic Acids Research.

[B47] Edgar RC (2004). MUSCLE: a multiple sequence alignment method with reduced time and space complexity. BMC Bioinformatics.

[B48] EBI home. http://www.ebi.ac.uk.

[B49] Blast2 site. http://www.ncbi.nlm.nih.gov/blast/bl2seq/wblast2.cgi.

