# Supplementary material for: The RHNumtS compilation: Features and bioinformatics approaches to locate and quantify Human NumtS
Source: BMC Genomics. 2008 Jun 3;9:267. doi: 10.1186/1471-2164-9-267 (PMC2447851; doi:10.1186/1471-2164-9-267)
Supplement: Additional file 4 — Multi-alignments of sequenced NumtS from 4 different phylo-geographic samples. The nucleotide multi-alignments of the amplified (fig 2a in the manuscript) and sequenced NumtS 87, 122 and 41–54 of individuals coming from Europe, Japan, Latin America and North Africa belonging respectively to haplogroups H2b, G1a1a, I3a and L2a1c1 compared with the NumtS sequence as it can be extracted from the Human Genome build36.2 through the UCSC genome browser (hg18 release) and the sequences of the corresponding mitochondrial region for the same samples, are reported. Multi-alignments of NumtS 87 and 122 include the rCRS sequence (accession number J01415.2 in GenBank) also, thus allowing the exact localization of the variant sites respect to the universally used human mitochondrial reference sequence. As far as NumtS 41-54 the reference sequence has been added to NumtS 43 only in a distinctly reported multialignment. The conservation of NumtS is evident from the multialignment among the different subjects, although heterozygous sites can be observed (nucleotide ambiguity letter such as Y for C/T, R for A/G etc.). Each multi-alignment refers to the nuclear region (from Chromosome start to Chromosome end), as reported in additional file 1. The NumtS sequences produced in our validation experiments are named with a code defined by the NumtS code and the haplogroup of the sample. The corresponding mtDNA regions are coded as mt, followed by the haplogroup code. The reference sequence for 122 NumtS is extracted from Celera genome chromosome 9 (CM00260). Sequencing of L2a1c1 122 NumtS, H2b and L2a1c1 41, 42 and 54 NumtS failed. [file 1471-2164-9-267-S4.pdf]

NumtS 87

```
           1
n87_H2b    CTGACTACTC CTACCATCAT GACCCCTAGC CATAATATGA TTTATCTCCA CACTAGCAGA AACTAACCGA
n87_L2a1c1 CTGACTACTC CTACCATCAT GACCCCTAGC CATAATATGA TTTATCTCCA CACTAGCAGA AACTAACCGA
n87_G1a1a  CTGACTACTC CTACCATCAT GACCCCTAGC CATAATATGA TTTATCTCCA CACTAGCAGA AACTAACCGA
n87_I3a    CTGACTACTC CTACCRTCAT GACCCCTAGC CATAATATGA TTTATCTCCA CACTAGCAGA AACTAACCGA
n87_hg18   CTGACTACTC CTACCATCAT GACCCCTAGC CATAATATGA TTTATCTCCA CACTAGCAGA AACTAACCGA
mt_H2b     CTGATTACTC CTGCCATCAT GACCCTTGGC CATAATATGA TTTATCTCCA CACTAGCAGA GACCAACCGA
mt_L2a1c1  CTGATTACTC CTGCCATCAT GACCCTTGGC CATAATATGA TTTATCTCCA CACTAGCAGA GACCAACCGA
mt_I3a     CTGATTACTC CTGCCATCAT GACCCTTGGC CATAATATGA TTTATCTCCA CACTAGCAGA GACCAACCGA
mt_G1a1a   CTGATTACTC CTGCCATCAT GACCCTTGGC CATAATATGA TTTATCTCCA CACTAGCAGA GACCAACCGA

          71
n87_H2b    GCCCCCTTCG ATCTTACAGA AGGAGAATCA GAATTAGTCT CAGGCTTCAA TATCGAATAT GCCGCAGGCC
n87_L2a1c1 GCCCCCTTCG ATCTTACAGA AGGAGAATCA GAATTAGTCT CAGGCTTCAA TATCGAATAT GCCGCAGGCC
n87_G1a1a  GCCCCCTTCG ATCTTACAGA AGGAGAATCA GAATTAGTCT CAGGCTTCAA TATCGAATAT GCCGCAGGCC
n87_I3a    GCCCCCTTCG ATCTTACAGA AGGAGAATCA GAATTAGTCT CAGGCTTCAA TATCGAATAT GCCGCAGGCC
n87_hg18   GCCCCCTTCG ATCTTACAGA AGGAGAATCA GAATTAGTCT CAGGCTTCAA TATCGAATAT GCCGCAGGCC
mt_H2b     ACCCCCTTCG ACCTTGCCGA AGGGGAGTCC GAACTAGTCT CAGGCTTCAA CATCGAATAC GCCGCAGGCC
mt_L2a1c1  ACCCCCTTCG ACCTTGCCGA AGGGGAATCC GAACTAGTCT CAGGCTTCAA CATCGAATAC GCCGCAGGCC
mt_I3a     ACCCCCTTCG ACCTTGCCGA AGGGGAGTCC GAACTAGTCT CAGGCTTCAA CATCGAATAC GCCGCAGGCC
mt_G1a1a   ACCCCCTTCG ACCTTGCCGA AGGGGAGTCC GAACTAGTCT CAGGCTTCAA CATCGAATAC GCCGCAGGCC

         141
n87_H2b    CATTTGCCCT CTTCTTTATA GCTGAGTATA TAAACATTAT TATAATAAAT GCCCTAACTG CTACAATTTT
n87_L2a1c1 CATTTGCCCT CTTCTTTATA GCTGAGTATA TAAACATTAT TATAATAAAT GCCCTAACTG CTACAATTTT
n87_G1a1a  CATTTGCCCT CTTCTTTATA GCTGAGTATA TAAACATTAT TATAATAAAT GCCCTAACTG CTACAATTTT
n87_I3a    CATTTGCCCT CTTCTTTATA GCTGAGTATA TAAACATTAT TATAATAAAT GCCCTAACTG CTACAATTTT
n87_hg18   CATTTGCCCT CTTCTTTATA GCTGAGTATA TAAACATTAT TATAATAAAT GCCCTAACTG CTACAATTTT
mt_H2b     CCTTCGCCCT ATTCTTCATA GCCGAATACA CAAACATTAT TATAATAAAC ACCCTCACCA CTACAATCTT
mt_L2a1c1  CCTTCGCCCT ATTCTTCATA GCCGAATACA CAAACATTAT TATAATAAAC ACCCTCACCA CTACAATCTT
mt_I3a     CCTTCGCCCT ATTCTTCATA GCCGAATACA CAAACATTAT TATAATAAAC ACCCTCACCA CTACAATCTT
mt_G1a1a   CCTTCGCCCT ATTCTTCATA GCCGAATACA CAAACATTAT TATAATAAAC ACCCTCACCA CTACAATCTT

         211
n87_H2b    TCTAGGAGCA CTATGCACTA TTTATTCACC AGAACTCTAT ACGATATATT TTACTATTAA AACTGTCCTC
n87_L2a1c1 TCTAGGAGCA CTATGCACTA TTTATTCACC AGAACTCTAT ACGATATATT TTACTATTAA AACTGTCCTC
n87_G1a1a  TCTAGGAGCA CTATGCACTA TTTATTCACC AGAACTCTAT ACGATATATT TTACTATTAA AACTGTCCTC
n87_I3a    TCTAGGAGCA CTATGCACTA TTTATTCACC AGAACTCTAT ACGATATATT TTACTATTAA AACTGTCCTC
n87_hg18   TCTAGGAGCA CTATGCACTA TTTATTCACC AGAACTCTAT ACGATATATT TTACTATTAA AACTGTCCTC
mt_H2b     CCTAGGAACA ACATATGACG CACTCTCCCC TGAACTCTAC ACAACATATT TTGTCACCAA GACCCTACTT
mt_L2a1c1  CCTAGGAACA ACATATGACG CACTCTCCCC TGAACTCTAC ACAACATATT TTGTCACCAA GACCCTACTT
mt_I3a     CCTAGGAACA ACATATGACG CACTCTCCCC TGAACTCTAC ACAACATATT TTGTCACCAA GACCCTACTT
mt_G1a1a   CCTAGGAACA ACATATGACG CACTCTCCCC TGAACTCTAC ACAACATATT TTGTCACCAA GACCCTACTT

         281
n87_H2b    CTAACCTCCC TATTTTTATG AATTCAAACA GCATACCCCC AATTCTGCTA CGATCAGCTT ATATACCTTT
n87_L2a1c1 CTAACCTCCC TATTTTTATG AATTCAAACA GCATACCCCC AATTCYGCTA CGATCAGCTT ATATACCTTT
n87_G1a1a  CTAACCTCCC TATTTTTATG AATTCAAACA GCATACCCCC AATTCTGCTA CGATCAGCTT ATATACCTTT
n87_I3a    CTAACCTCCC TATTTTTATG AATTCAAACA GCATACCCCC AATTCYGCTA CGATCAGCTT ATATACCTTT
n87_hg18   CTAACCTCCC TATTTTTATG AATTCAAACA GCATACCCCC AATTCTGCTA CGATCAGCTT ATATACCTTT
mt_H2b     CTAACCTCCC TGTTCTTATG AATTCGAACA GCATACCCCC GATTCCGCTA CGACCAACTC ATACACCTCC
mt_L2a1c1  CTGACCTCCC TGTTCTTATG AATTCGAACA GCATACCCCC GATTCCGCTA CGACCAACTT ATACACCTCC
mt_I3a     CTAACCTCCC TGTTCTTATG AATTCGAACA GCATACCCCC GATTCCGCTA CGACCAACTC ATACACCTCC
mt_G1a1a   CTAACCTCCC TGTTCTTATG AATTCGAACA GCATACCCCC GATTCCGCTA CGACCAACTC ATACACCTCC

         351
n87_H2b    TATGAAAAAA CTGTCTGCCA CTCACACTGG CATTTTTCAT ATGGTATATT TCAATACCCG TCACAATCTC
n87_L2a1c1 TATGAAAAAA CTGTCTGCCA CTCACACTGG CATTTTTCAT ATGGTATATT TCAATACCCG TCACAATCTC
n87_G1a1a  TATGAAAAAA CTGTCTGCCA CTCACACTGG CATTTTTCAT ATGGTATATT TCAATACCCG TCACAATCTC
n87_I3a    TATGAAAAAA CTGTCTGCCA CTCACACTGG CATTTTTCAT ATGGTATATT TCAATACCCG TCACAATCTC
n87_hg18   TATGAAAAAA CTGTCTGCCA CTCACACTGG CATTTTTCAT ATGGTATATT TCAATACCCG TCACAATCTC
mt_H2b     TATGAAAAAA CTTCCTACCA CTCACCCTAG CATTACTTAT ATGATATGTC TCCATACCCA TTACAATCTC
mt_L2a1c1  TATGAAAAAA CTTCCTACCA CTCACCCTAG CATTACTTAT ATGATATGTC TCCATACCCA TTACAATCTC
mt_I3a     TATGAAAAAA CTTCCTACCA CTCACCCTAG CATTACTTAT ATGATATGTC TCCATACCCA TTACAATCTC
mt_G1a1a   TATGAAAAAA CTTCCTACCA CTCACCCTAG CATTACTTAT ATGATATGTC TCCATACCCA TTACAATCTC

         421
n87_H2b    CAATATTCCC CC-CAAACCT AAGAAATATG TCTGATAAAA GAGTTACTTT GATAGAGTAA ATAATAG
n87_L2a1c1 CAATATTCCC CC-CAAACCT AAGAAATATG TCTGATAAAA GAGTTACTTT GATAGAGTAA ATAATAG
n87_G1a1a  CAATATTCCC CC-CAAACCT AAGAAATATG TCTGATAAAA GAGTTACTTT GATAGAGTAA ATAATAG
n87_I3a    CAATATTCCC CC-CAAACCT AAGAAATATG TCTGATAAAA GAGTTACTTT GATAGAGTAA ATAATAG
n87_hg18   CAATATTCCC CC-CAAACCT AAGAAATATG TCTGATAAAA GAGTTACTTT GATAGAGTAA ATAAT--
mt_H2b     CAGCATTCCC CCTCAAACCT AAGAAATATG TCTGATAAAA GAGTTACTTT GATAGAGTAA ATAATAG
mt_L2a1c1  CAGCATTCCC CCTCAAACCT AAGAAATATG TCTGATAAAA GAGTTACTTT GATAGAGTAA ATAATAG
mt_I3a     CAGCATTCCC CCTCAAACCT AAGAAATATG TCTGATAAAA GAGTTACTTT GATAGAGTAA ATAATAG
mt_G1a1a   CAGCATTCCC CCTCAAACCT AAGAAATATG TCTGATAAAA GAGTTACTTT GATAGAGTAA ATAATAG
```

NumtS 122

```
                 1
n122_H2b         --AGCTGCTT CTTTGAATTT ACAATTCAAC ATGAAAATCA CCTCGGGACT GGTAAAAACA GGCCTTGACC
n122_L2a1c1      GAAGCTGCTT CTTTGAATTT ACAATTCAAC ATGAAAATCA CCTCGGGACT GGTAAAAACA GGCCTTGACC
n122_G1a1a       GAAGCTGCTT CTTTGAATTT ACAATTCAAC ATGAAAATCA CCTCGGGACT GGTAAAAACA GGCCTTGACC
n122_Celera      GAAGCTGCTT CTTTGAATTT ACAATTCAAC ATGAAAATCA CCTCGGGACT GGTAAAAACA GGCCTTGACC
mt_H2b           GAAGCTGCTT CTTCGAATTT GCAATTCAAC ATGAAAATCA CCTCGGAGCT GGTAAAAAGA GGCC-TAACC
mt_L2a1c1        GAAGCTGCTT CTTCGAATTT GCAATTCAAT ATGAAAATCA CCTCGGAGCT GGTAAAAAGA GGCC-TAACC
mt_G1a1a         GAAGCTGCTT CTTCGAATTT GCAATTCAAT ATGAAAATCA CCTCGGAGCT GGTAAAAAGA GGCC-TAACC

                71
n122_H2b         TCTGTTTTTA GATGTACAGT CTAATGCCCT ACTCAGTCAT TTTACCCTTT TTTCTCACTT AATTTATGTT
n122_L2a1c1      TCTGTTTTTA GATGTACAGT CTAATGCCCT ACTCAGTCAT TTTACCCTTT TTTCTCACTT AATTTATGTT
n122_G1a1a       TCTGTTTTTA GATGTACAGT CTAATGCCCT ACTCAGTCAT TTTACCCTTT TTTCTCACTT AATTTATGTT
n122_Celera      TCTGTTTTTA GATGTACAGT CTAATGCCCT ACTCAGTCAT TTTACCCTTT TTTCTCACTT AATTTATGTT
mt_H2b           CCTGTCTTTA GATTTACAGT CCAATGCTTC ACTCAGCCAT TTTACCTCAC CCCC------ -ACTGATGTT
mt_L2a1c1        CCTGTCTTTA GATTTACAGT CCAATGCTTC ACTCAGCCAT TTTACCTCAC CCCC------ -ACTGATGTT
mt_G1a1a         CCTGTCTTTA GATTTACAGT CCAATGCTTC ACTCAGCCAT TTTACCTCAC CCC------- -ACTGATGTT

               141
n122_H2b         GGCTGACAGT TGACTATTCT CAACCAACCA TAAAGATATC GGGACATTAT ATTTATTATT TGGCACATGA
n122_L2a1c1      GGCTGACAGT TGACTATTCT CAACCAACCA TAAAGATATC GGGACATTAT ATTTATTATT TGGCACATGA
n122_G1a1a       GGCTGACAGT TGACTATTCT CAACCAACCA TAAAGATATC GGGACATTAT ATTTATTATT TGGCACATGA
n122_Celera      GGCTGACAGT TGACTATTCT CAACCAACCA TAAAGATATC GGGACATTAT ATTTATTATT TGGCACATGA
mt_H2b           CGCCGACCGT TGACTATTCT CTACAAACCA CAAAGACATT GGAACACTAT ACCTATTATT CGGCGCATGA
mt_L2a1c1        CGCCGACCGT TGACTATTCT CTACAAACCA CAAAGACATT GGAACACTAT ACCTATTATT CGGCGCATGA
mt_G1a1a         CGCCGACCGT TGACTATTCT CTACAAACCA CAAAGACATT GGAACACTAT ACCTATTATT CGGCGCATGA

               211
n122_H2b         GCAGGGATAG TCAGAACAGC TTTAAG---C CTTATTCGAG CTGAATT--- ---------- ---CTACTAG
n122_L2a1c1      GCAGGGATAG TCAGAACAGC TTTAAG---C CTTATTCGAG CTGAATT--- ---------- ---CTACTAG
n122_G1a1a       GCAGGGATAG TCAGAACAGC TTTAAG---C CTTATTCGAG CTGAATT--- ---------- ---CTACTAG
n122_Celera      GCAGGGATAG TCAGAACAGC TTTAAG---C CTTATTCGAG CTGAATT--- ---------- ---CTACTAG
mt_H2b           GCTGGAGTCC TAGGCACAGC TCTAAGCCTC CTTATTCGAG CCGAGCTGGG CCAGCCAGGC AACCTTCTAG
mt_L2a1c1        GCTGGAGTCC TAGGCACAGC TCTAAGCCTC CTTATTCGAG CCGAGCTGGG CCAGCCAGGC AACCTTCTAG
mt_G1a1a         GCTGGAGTCC TAGGCACAGC TCTAAGCCTC CTTATTCGAG CCGAGCTGGG CCAGCCAGGC AACCTTCTAG

               281
n122_H2b         ---ATGATCA AATTTG---- --TCTTGTTA TAGCCTATGC ATTTGTCATA ATTTTCTTTA TAGTAATAC-
n122_L2a1c1      ---ATGATCA AATTTG---- --TCTTGTTA TAGCCTATGC ATTTGTCATA ATTTTCTTTA TAGTAATAC-
n122_G1a1a       ---ATGATCA AATTTG---- --TCTTGTTA TAGCCTATGC ATTTGTCATA ATTTTCTTTA TAGTAATAC-
n122_Celera      ---ATGATCA AATTTG---- --TCTTGTTA TAGCCTATGC ATTTGTCATA ATTTTCTTTA TAGTAATAC-
mt_H2b           GTAACGACCA CATCTACAAC GTTATCGTCA CAGCCCATGC ATTTGTAATA ATCTTCTTCA TAGTAATACC
mt_L2a1c1        GTAACGACCA CATCTACAAC GTTATCGTCA CAGCCCATGC ATTTGTAATA ATCTTCTTCA TAGTAATACC
mt_G1a1a         GTAACGACCA CATCTACAAC GTTATCGTCA CAGCCCATGC ATTTGTAATA ATCTTCTTCA TAGTAATACC

               351
n122_H2b         ---TATAATT GTAGGTCTTG GCAACTGATT AGTCCCCCY- ATAATTGGCG CCCCCGATAT AGCATTTCTC
n122_L2a1c1      ---TATAATT GTAGGTCTTG GCAACTGATT AGTCCCCCTG ATAATTGGCG CCCCCGATAT AGCATTTCTC
n122_G1a1a       ---TATAATT GTAGGTCTTG GCAACTGATT AGTCCCCCYG ATAATTGGCG CCCCCGATAT AGCATTTCTC
n122_Celera      ---TATAATT GTAGGTCTTG GCAACTGATT AGTCCCCCT- ATAATTGGCG CCCCCGATAT AGCATTTCTC
mt_H2b           CATCATAATC GGAGGCTTTG GCAACTGACT AGTTCCCCTA ATAATCGGTG CCCCCGATAT GGCGTTTCCC
mt_L2a1c1        CATCATAATC GGAGGCTTTG GCAACTGACT AGTTCCCCTA ATAATCGGTG CCCCCGATAT GGCGTTTCCC
mt_G1a1a         CATCATAATC GGAGGCTTTG GCAACTGACT AGTTCCCCTA ATAATCGGTG CCCCCGATAT GGCGTTTCCC

               421
n122_H2b         TGCATAAATA ATATGAGCTT CTGACTCCTC CCACCCTCCT TCCTATTATA ACTTGCATCC ACTATAGTAG
n122_L2a1c1      TGCATAAATA ATATGAGCTT CTGACTCCTC CCACCCTCCT TCCTATTATA ACTTGCATCC ACTATAGTAG
n122_G1a1a       TGCATAAATA ATATGAGCTT CTGACTCCTC CCACCCTCCT TCCTATTATA ACTTGCATCC ACTATAGTAG
n122_Celera      TGCATAAATA ATATGAGCTT CTGACTCCTC CCACCCTCCT TCCTATTATA ACTTGCATCC ACTATAGTAG
mt_H2b           CGCATAAACA ACATAAGCTT CTGACTCTTA CCTCCCTCTC TCCTACTCCT GCTCGCATCT GCTATAGTGG
mt_L2a1c1        CGCATAAACA ACATAAGCTT CTGACTCTTA CCTCCCTCTC TCCTACTCCT GCTCGCATCT GCTATAGTGG
mt_G1a1a         CGCATAAACA ACATAAGCTT CTGACTCTTA CCTCCCTCTC TCCTACTCCT GCTCGCATCT GCTATAGTGG

               491
n122_H2b         AAGCCGGCAC TGGAACCGGC TGAACAGTCT CTCCTCCCTT AGCAGRAAAC CTAACACATG CAGGCGCCTC
n122_L2a1c1      AAGCCGGCAC TGGAACCGGC TGAACAGTCT CTCCTCCCTT AGCAGGAAAC CTAACACATG CAGGCGCCTC
n122_G1a1a       AAGCCGGCAC TGGAACCGGC TGAACAGTCT CTCCTCCCTT AGCAGGAAAC CTAACACATG CAGGCGCCTC
n122_Celera      AAGCCGGCAC TGGAACCGGC TGAACAGTCT CTCCTCCCTT AGCAGGAAAC CTAACACATG CAGGCGCCTC
mt_H2b           AGGCCGGAGC AGGAACAGGT TGAACAGTCT ACCCTCCCTT AGCAGGGAAC TACTCCCACC CTGGAGCCTC
mt_L2a1c1        AGGCCGGAGC AGGAACAGGT TGAACAGTCT ACCCTCCCTT AGCAGGGAAC TACTCCCACC CTGGAGCCTC
mt_G1a1a         AGGCCGGAGC AGGAACAGGT TGAACAGTCT ACCCTCCCTT AGCAGGGAAC TACTCCCACC CTGGAGCCTC
```

```
               561
n122_H2b       TGTAGATTTC ACTRTCTTTT CACTCCACTT GGCAGGTGTT TCTTCTACTT CAGAGGCTAT TAACTTTATT
n122_L2a1c1    TGTAGATTTC ACTGTCTTTT CACTCCACTT GGCAGGTGTT TCTTCTACTT CAGAGGCTAT TAACTTTATT
n122_G1a1a     TGTAGATTTC ACTGTCTTTT CACTCCACTT GGCAGGTGTT TCTTCTACTT CAGAGGCTAT TAACTTTATT
n122_Celera    TGTAGATTTC ACTGTCTTTT CACTCCACTT GGCAGGTGTT TCTTCTACTT CAGAGGCTAT TAACTTTATT
mt_H2b         CGTAGACCTA ACCATCTTCT CCTTACACCT AGCAGGTGTC TCCTCTATCT TAGGGGCCAT CAATTTCATC
mt_L2a1c1      CGTAGACCTA ACCATCTTCT CCTTACACCT AGCAGGTGTC TCCTCTATCT TAGGGGCCAT CAATTTCATC
mt_G1a1a       CGTAGACCTA ACCATCTTCT CCTTACACCT AGCAGGTGTC TCCTCTATCT TAGGGGCCAT CAATTTCATC

               631
n122_H2b       ATCACAATTG TTAATATAAA ACCCCCAGCC ATGTCCCAAT ATCACACACC CCTCTTCATC TGATTAGTCC
n122_L2a1c1    ATCACAATTG TTAATATAAA ACCCCCAGCC ATGTCCCAAT ATCACACACC CCTCTTCATC TGATTAGTCC
n122_G1a1a     ATCACAATTG TTAATATAAA ACCCCCAGCC ATGTCCCAAT ATCACACACC CCTCTTCATC TGATTAGTCC
n122_Celera    ATCACAATTG TTAATATAAA ACCCCCAGCC ATGTCCCAAT ATCACACACC CCTCTTCATC TGATTAGTCC
mt_H2b         ACAACAATTA TCAATATAAA ACCCCCTGCC ATAACCCAAT ACCAAACGCC CCTCTTCGTC TGATCCGTCC
mt_L2a1c1      ACAACAATTA TCAATATAAA ACCCCCTGCC ATAACCCAAT ACCAAACGCC CCTCTTCGTC TGATCCGTCC
mt_G1a1a       ACAACAATTA TCAATATAAA ACCCCCTGCC ATAACCCAAT ACCAAACGCC CCTCTTCGTC TGATCCGTCC

               701
n122_H2b       TAATTACAGC AGTTCTTCTA CTCCTTTGTC TACGAGTCCT AGCCTCCGGC ATCACTATA-
n122_L2a1c1    TAATTACAGC AGTTCTTCTA CTCCTTTGTC TACGAGTCCT AGCCTCCGGC ATCACTATA-
n122_G1a1a     TAATTACAGC AGTGCTTCTA CTCCTTTGTC TACGAGTCCT AGCCTCCGGC ATCACTATA-
n122_Celera    TAATTACAGC AGTTCTTCTA CTCCTTTGTC TACGAGTCCT AGCCTCCGGC ATCACTATA-
mt_H2b         TAATCACAGC AGTCCTACTT CTCCTATCTC TCCCAGTCCT AGCTGCTGGC ATCACTATAC
mt_L2a1c1      TAATCACAGC AGTCCTACTT CTCCTATCTC TCCCAGTCCT AGCTGCTGGC ATCACTATAC
mt_G1a1a       TAATCACAGC AGTCCTACTT CTCCTATCTC TCCCAGTCCT AGCTGCTGGC ATCACTATAC
```

NumtSs 41-54

```
              1
n41_54_H2b    ---------- ---------- ---------- ---------- ---------- ---------- ----------
n41_54_L2a1c1 ---------- ---------- ---------- ---------- ---------- ---------- ----------
n41_54_G1a1a  GAAGGC-CAA CCCCAGTCTC AGTTCTACTC CACTCCAGCA CTATAGTTGT AGCAGGTGTT TCAAGGTCCA
n41_54_I3a    GAAGGCWCAA CCCCAGTYTC AGTTCTACTC CACTCCAGCA CTATAGTTGT AGCAGGTGTT TGAAGGTCCA
n41_54_hg18   GAAGGCCCAA CCCCAGTTTC AGTTCTACTC CACTCCAGCA CTATAGTTGT AGCAGGTGTT TCAAGGTCCA
mt_H2b        GAAGGCCCCA CCCCAGTCTC AGCCCTACTC CACTCAAGCA CTATAGTTGT AGCAGG---- -GAAGGCCCC
mt_L2a1c1     GAAGGCCCCA CCCCAGTCTC AGCCCTACTC CACTCAAGCA CTATAGTTGT AGCAGG---- -GAAGGCCCC
mt_G1a1a      GAAGGCCCCA CCCCAGTCTC AGCCCTACTC CACTCAAGCA CTATAGTTGT AGCAGG---- -GAAGGCCCC
mt_I3a        GAAGGCCCCA CCCCAGTCTC AGCCCTACTC CACTCAAGCA CTATAGTTGT AGCAGG---- -GAAGGCCCC

             71
n41_54_H2b    ---------- ---------- ---------- ---------- ---------- ---------- ----------
n41_54_L2a1c1 ---------- ---------- ---------- ---------- ---------- ---------- ----------
n41_54_G1a1a  ASCCCAGTCT CAGCCCTGCT CCGCTCCAGC ACTATAGTTG TAGCAGGTGT TTGAAGGCCC AACCCCAGTC
n41_54_I3a    ACCCCAGTCT CAGCCCTGCT CCGCTCCAGC ACTATAGTTG TAGCAGGTGT TTGAAGGCCC AACCCCAGTC
n41_54_hg18   AGCCCAGTCT CAGCCCTGCT CCGCTCCAGC ACTATAGTTG TAGCAGGTGT TTGAAGGCCC AACCCCAGTC
mt_H2b        ACCCCAGTCT CAGCCCTACT CCACTCAAGC ACTATAGTTG TAGCAGG--- --GAAGGCCC CACCCCAGTC
mt_L2a1c1     ACCCCAGTCT CAGCCCTACT CCACTCAAGC ACTATAGTTG TAGCAGG--- --GAAGGCCC CACCCCAGTC
mt_G1a1a      ACCCCAGTCT CAGCCCTACT CCACTCAAGC ACTATAGTTG TAGCAGG--- --GAAGGCCC CACCCCAGTC
mt_I3a        ACCCCAGTCT CAGCCCTACT CCACTCAAGC ACTATAGTTG TAGCAGG--- --GAAGGCCC CACCCCAGTC

            141
n41_54_H2b    ---------- ---------- ---------- ---------- ---------- ---------- ----------
n41_54_L2a1c1 ---------- ---------- ---------- ---------- ---------- ---------- ----------
n41_54_G1a1a  TCAGCCCAGC TCCACTCCAG CACTAGAGTT GTAGCAGGTG TTTGAAGGCC CAACCCCAGT CTCAGCCCTG
n41_54_I3a    TCAGCCCAGC TCCACTCCAG CACTAAAGTT GTAGCAGGTG TTTGAAGGCC CAACCCCAGT CTCAGCCCTG
n41_54_hg18   TCAGCCCAGC TCCACTCCAG CACTATAGTT GTAGCAGGTG TTTGAAGGCC CAACCCCAGT CTCAGCCCTG
mt_H2b        TCAGCCCTAC TCCACTCAAG CACTATAGTT GTAGCAGG-- ---GAAGGCC CCACCCCAGT CTCAGCCCTA
mt_L2a1c1     TCAGCCCTAC TCCACTCAAG CACTATAGTT GTAGCAGG-- ---GAAGGCC CCACCCCAGT CTCAGCCCTA
mt_G1a1a      TCAGCCCTAC TCCACTCAAG CACTATAGTT GTAGCAGG-- ---GAAGGCC CCACCCCAGT CTCAGCCCTA
mt_I3a        TCAGCCCTAC TCCACTCAAG CACTATAGTT GTAGCAGG-- ---GAAGGCC CCACCCCAGT CTCAGCCCTA

            211
n41_54_H2b    ---------- ---------- ---------- ----GAAGGC CCAACCCCAG TCTCAGCCCT GCTCCACTCC
n41_54_L2a1c1 ---------- ---------- ---------- ----GAAGGC CCAACCCCAG TTTCAGCTCT GCTCCACTCC
n41_54_G1a1a  CTCCACTCCA GCACTAAAGT TGT-GCAG-T GTTTGAAGGT CCAACCCCAG TCTCAGCCCT GCTCCGCTCC
n41_54_I3a    CTCCGCTCCA GCACTATAGT TGTAGCAGGT GTTTGAAGGT RCAACCCCAG TCTCAGCCCT GCTCCACTCC
n41_54_hg18   CTCCGCTCCA GCACTATAGT TGTAGCAGGT GTTTGAAGGT CCAAGCCCAG TCTCAGCCCT GCTCCACTCC
mt_H2b        CTCCACTCAA GCACTATAGT TGTAGCAGG- ----GAAGGC CCCACCCCAG TCTCAGCCCT ACTCCACTCA
mt_L2a1c1     CTCCACTCAA GCACTATAGT TGTAGCAGG- ----GAAGGC CCCACCCCAG TCTCAGCCCT ACTCCACTCA
mt_G1a1a      CTCCACTCAA GCACTATAGT TGTAGCAGG- ----GAAGGC CCCACCCCAG TCTCAGCCCT ACTCCACTCA
mt_I3a        CTCCACTCAA GCACTATAGT TGTAGCAGG- ----GAAGGC CCCACCCCAG TCTCAGCCCT ACTCCACTCA

            281
n41_54_H2b    AGCACTATAG TTGTAGCAGG TGTTTGAAGG CCCAACCCCA GTCTCAGCCC TGCTCCRCTC CAGCACTATA
n41_54_L2a1c1 AGCACTATAG TTGTAGCAGG TGTTTGAAGG CCCAAGCCCA GTCTCAGCCC TGCTCCGCTC CAGCACTATA
n41_54_G1a1a  AGCACTAWAG TTGTAGCAGG TGTTTGAAGG CCCAACCCCA GTCTCAGCCC TGCTCCRCTC CAGCACTATA
n41_54_I3a    AGCACTATAG TTGTAGCAGG WGTTTGAAGG CCCAACCCCA GTCTCAGCCC TGCTCCGCTC CAGCACTATA
n41_54_hg18   AGCACTATAG TTGTAACAGG TGTTTGAAGG CCCAACCCCA GTCTCAGCCC TGCTCCGCTC CAGCACTATA
mt_H2b        AGCACTATAG TTGTAGCAGG -----GAAGG CCCCACCCCA GTCTCAGCCC TACTCCACTC AAGCACTATA
mt_L2a1c1     AGCACTATAG TTGTAGCAGG -----GAAGG CCCCACCCCA GTCTCAGCCC TACTCCACTC AAGCACTATA
mt_G1a1a      AGCACTATAG TTGTAGCAGG -----GAAGG CCCCACCCCA GTCTCAGCCC TACTCCACTC AAGCACTATA
mt_I3a        AGCACTATAG TTGTAGCAGG -----GAAGG CCCCACCCCA GTCTCAGCCC TACTCCACTC AAGCACTATA

            351
n41_54_H2b    GTTGTAG-CA GGTGTTTGAA GGTCCAACCC CAGTCTCAGC CCTGCTCCRC TCCAGCACTA TAGTTGTAGC
n41_54_L2a1c1 GTTGTAGCCA GGTGTTTGAA GGCCCAACCC CAGTCTCAGC CCTGCTCCGC TCCAGCACTA TAGTTGTAGC
n41_54_G1a1a  GTTGTAG-CA GGTGTTTGAA GGTCCAACCC CAGTCTCAGC CCTGCTCCGC TCCAGCACTA TAGTTGTAGC
n41_54_I3a    GTTGTAG-CA GGTGTTTGAA GGTCC-ACCC CAGTCTCAGC CCTGCTCCGC TCCAGCACTA TAGTTGTAGC
n41_54_hg18   GTTGTAG-CA GGTGTTTGAA GGTCCAACCC CAGTCTCAGC CCAGCTCCAC TCCAGCACTA TAGTTGTAGC
mt_H2b        GTTGTAG-CA GG-----GAA GGCCCCACCC CAGTCTCAGC CCTACTCCAC TCAAGCACTA TAGTTGTAGC
mt_L2a1c1     GTTGTAG-CA GG-----GAA GGCCCCACCC CAGTCTCAGC CCTACTCCAC TCAAGCACTA TAGTTGTAGC
mt_G1a1a      GTTGTAG-CA GG-----GAA GGCCCCACCC CAGTCTCAGC CCTACTCCAC TCAAGCACTA TAGTTGTAGC
mt_I3a        GTTGTAG-CA GG-----GAA GGCCCCACCC CAGTCTCAGC CCTACTCCAC TCAAGCACTA TAGTTGTAGC

            421
n41_54_H2b    AGGTGTTTCG AAGGCACAAC CCCAGTCTCA GCCCTGCTCC RCTCCAGCAC TATAGTTGTA GCAGGTGTTT
n41_54_L2a1c1 AGGTGTTT-G AAGGCCCAAC CCCAGTCTCA GCCCTGCTCC GCTCCAGCAC TATAGTTGTA GCAGGTGTTT
n41_54_G1a1a  AGGTGTTT-G AAGGCCCAAC CCCAGTCTCA GCCCTGCTCC GCTCCAGCAC TATAGTTGTA GCAGGTGTTT
n41_54_I3a    AGGTGTTT-G AAGGCCCAAC CCCAGTCTCA GCCCTGCTCC RCTCCAGCAC TATAGTTGTA GCAGGTGTTT
n41_54_hg18   AGGTGTTT-G AAGGCCCAAC CCCAGTCTCA GCCCTGCTCC GCTCCAGCAC TATAGTTGTA GCAGGTGTTT
mt_H2b        AGG------G AAGGCCCCAC CCCAGTCTCA GCCCTACTCC ACTCAAGCAC TATAGTTGTA GCAGG-----
mt_L2a1c1     AGG------G AAGGCCCCAC CCCAGTCTCA GCCCTACTCC ACTCAAGCAC TATAGTTGTA GCAGG-----
mt_G1a1a      AGGV-----G AAGGCCCCAC CCCAGTCTCA GCCCTACTCC ACTCAAGCAC TATAGTTGTA GCAGG-----
mt_I3a        AGG------G AAGGCCCCAC CCCAGTCTCA GCCCTACTCC ACTCAAGCAC TATAGTTGTA GCAGG-----
```

```
                491
n41_54_H2b      GAAGGTCCCA ACCCCAGTCT CAGCCCTGCT CCRCTCCAGC ACTATAGTTG TAGCAGGTGT TTGAAGGCCC
n41_54_L2a1c1   KAAGGTCC-A ACCCCAGTCT CAGCCCTGCT CCGCTCCAGC ACTATAGTTG TAGCAGGTGT TTGAAGGCCC
n41_54_G1a1a    GAAGG-CCCA ACCCCAGTCT CAGCCCTGCT CCRCTCCAGC ACTATAGTTG TAGCAGGTGT TTGAAGGCCC
n41_54_I3a      GAAGG-CCCA ACCCCAGTCT CAGCCCTGCT CCGCTCCAGC ACTATAGTTG TAGCAGGTGT TTGAAGGCCC
n41_54_hg18     GAAGG-CCCA ACCCCAGTCT CAGCCCTGCT CCGCTCCAGC ACTATAGTTG TAGCAGGTGT TTGAAGGCCC
mt_H2b          GAAGG-CCCC ACCCCAGTCT CAGCCCTACT CCACTCAAGC ACTATAGTTG TAGCAGG--- --GAAGGCCC
mt_L2a1c1       GAAGG-CCCC ACCCCAGTCT CAGCCCTACT CCACTCAAGC ACTATAGTTG TAGCAGG--- --GAAGGCCC
mt_G1a1a        GAAGG-CCCC ACCCCAGTCT CAGCCCTACT CCACTCAAGC ACTATAGTTG TAGCAGG--- --GAAGGCCC
mt_I3a          GAAGG-CCCC ACCCCAGTCT CAGCCCTACT CCACTCAAGC ACTATAGTTG TAGCAGG--- --GAAGGCCC

                561
n41_54_H2b      AACCCCAGTC TCAGCCCTGC TCCRCTCCAG CACTATAGTT GTAGCAGGTG TTTGAAGGCC CAACCCCAGT
n41_54_L2a1c1   AACCCCAGTC TCAGCCCTGC TCCRCTCCAG CACTATAGTT GTAGCAGGTG TTTGAAGGCC CAACCCCAGT
n41_54_G1a1a    AACCCCAGTC TCAGCCCTGC TCCRCTCCAG CACTATAGTT GTAGCAGGTG TTTGAAGGCC CAACCCCAGT
n41_54_I3a      AACCCCAGTC TCAGCCCTGC TCCGCTCCAG CACTATAGTT GTAGCAGGTG TTTGAAGGCC CAACCCCAGT
n41_54_hg18     AACCCCAGTC TCAGCCCTGC TCCGCTCCAG CACTATAGTT GTAGCAGGTG TTTGAAGGTC CAACCCCAGT
mt_H2b          CACCCCAGTC TCAGCCCTAC TCCACTCAAG CACTATAGTT GTAGCAGG-- ---GAAGGCC CCACCCCAGT
mt_L2a1c1       CACCCCAGTC TCAGCCCTAC TCCACTCAAG CACTATAGTT GTAGCAGG-- ---GAAGGCC CCACCCCAGT
mt_G1a1a        CACCCCAGTC TCAGCCCTAC TCCACTCAAG CACTATAGTT GTAGCAGG-- ---GAAGGCC CCACCCCAGT
mt_I3a          CACCCCAGTC TCAGCCCTAC TCCACTCAAG CACTATAGTT GTAGCAGG-- ---GAAGGCC CCACCCCAGT

                631
n41_54_H2b      CTCAGCCCTG CTCCRCTCCA GCACTATAGT TGTAGCAGGT GTTTGAA-GG CCCAACCCCA GTCTCAGCCC
n41_54_L2a1c1   CTCAGCCCTG CTCCGCTCCA GCACTATAGT TGTAGCAGGT GTTTGAAGGG CCCAACCCCA GTCTCAGCCC
n41_54_G1a1a    CTCAGCCCAG CTCCRCTCCA GCACTATAGT TGTAGCAAGT GTTTGAA-GG CCCAACCCCA GTCTCAGCCC
n41_54_I3a      CTCAGCCCAG CTCCACTCCA GCACTATAGT TGTAGCAGGT GTTTGAA-GG CCCAACCCCA GTCTCAGCCC
n41_54_hg18     CTCAGCCCAG CTCCACTCCA GCACTATAGT TGTAGCAGGT GTTTGAA-GG CCCAACCCCA GTCTCAGCCC
mt_H2b          CTCAGCCCTA CTCCACTCAA GCACTATAGT TGTAGCAGG- ----GAA-GG CCCCACCCCA GTCTCAGCCC
mt_L2a1c1       CTCAGCCCTA CTCCACTCAA GCACTATAGT TGTAGCAGG- ----GAA-GG CCCCACCCCA GTCTCAGCCC
mt_G1a1a        CTCAGCCCTA CTCCACTCAA GCACTATAGT TGTAGCAGG- ----GAA-GG CCCCACCCCA GTCTCAGCCC
mt_I3a          CTCAGCCCTA CTCCACTCAA GCACTATAGT TGTAGCAGG- ----GAA-GG CCCCACCCCA GTCTCAGCCC

                701
n41_54_H2b      TGCTCCRCTC CAGCACTATA GTT-TAGCAG GTGTTTGAAG GCCCAACCCC AGTCTCAGCC CTGCTCCACT
n41_54_L2a1c1   TGCTCCRCTC CAGCACTATA GTTGTAGCAG GTGTTTGAAG GCCCAACCCC AGTCTCAGCC CTGCTCCGCT
n41_54_G1a1a    TGCTCCGCTC CAGCACTATA GTTGTAGCAG GTGTTTGAAG GTCCAAGCCC AGTCTCAGCC CTGCTCCACT
n41_54_I3a      TGCTCCGCTC CAGCACTATA GTTGTAGCAG GTGTTTGAAG GTCCAACCCC AGTCTCAGCC CTGCTCCACT
n41_54_hg18     TGCTCCGCTC CAGCACTATA GTTGTAGCAG GTGTTTGAAG GTCCAAGCCC AGTCTCAGCC CTGCTCCACT
mt_H2b          TACTCCACTC AAGCACTATA GTTGTAGCAG G-----GAAG GCCCCACCCC AGTCTCAGCC CTACTCCACT
mt_L2a1c1       TACTCCACTC AAGCACTATA GTTGTAGCAG G-----GAAG GCCCCACCCC AGTCTCAGCC CTACTCCACT
mt_G1a1a        TACTCCACTC AAGCACTATA GTTGTAGCAG G-----GAAG GCCCCACCCC AGTCTCAGCC CTACTCCACT
mt_I3a          TACTCCACTC AAGCACTATA GTTGTAGCAG G-----GAAG GCCCCACCCC AGTCTCAGCC CTACTCCACT

                771
n41_54_H2b      CCAGCACTAT AGTTGTAGCA GGTGTTT--- ---------- ---------- ---------- ----------
n41_54_L2a1c1   CCAGCACTAT AGTTGTAGCA GGTGTTT--- ---------- ---------- ---------- ----------
n41_54_G1a1a    CCAGCACTAT AGTTGTAGCA GGTGTTTGAA GGCCCAACCC CAGTCTCAGC CCTGCTCCGC TCC-AGCACT
n41_54_I3a      CCAGCACTAT AGTTGTAGCA GGTGTTTGAA GGCCCAACCC -AGTCTCAGC CCTGCTCCG- TCCCAGCACT
n41_54_hg18     CCAGCACTAT AGTTGTAGCA GGTGTTTGAA GGCCCAACCC CAGTCTCAGC CCTGCTCCGC TCC-AGCACT
mt_H2b          CAAGCACTAT AGTTGTAGCA GG-----GAA GGCCCCACCC CAGTCTCAGC CCTACTCCAC TC-AAGCACT
mt_L2a1c1       CAAGCACTAT AGTTGTAGCA GG-----GAA GGCCCCACCC CAGTCTCAGC CCTACTCCAC TC-AAGCACT
mt_G1a1a        CAAGCACTAT AGTTGTAGCA GG-----GAA GGCCCCACCC CAGTCTCAGC CCTACTCCAC TC-AAGCACT
mt_I3a          CAAGCACTAT AGTTGTAGCA GG-----GAA GGCCCCACCC CAGTCTCAGC CCTACTCCAC TC-AAGCACT

                841
n41_54_H2b      ---------- ----
n41_54_L2a1c1   ---------- ----
n41_54_G1a1a    ATAGTCATAG CAGG
n41_54_I3a      ATAGTCATAG CAGG
n41_54_hg18     ATAGTCATAG CAGG
mt_H2b          ATAGTTGTAG CAGG
mt_L2a1c1       ATAGTTGTAG CAGG
mt_G1a1a        ATAGTTGTAG CAGG
mt_I3a          ATAGTTGTAG CAGG
```
