# Supplementary material for: The RHNumtS compilation: Features and bioinformatics approaches to locate and quantify Human NumtS
Source: BMC Genomics. 2008 Jun 3;9:267. doi: 10.1186/1471-2164-9-267 (PMC2447851; doi:10.1186/1471-2164-9-267)
Supplement: Additional file 5 — Sequences of 22 NumtS from a European sample (haplogroup L2a1c1). Each sequence has been multi-aligned with the NumtS sequence as it can be extracted from the Human Genome build36.2 through the UCSC genome browser (hg18 release), the sequence of the corresponding mitochondrial region for the same sample and the rCRS sequence (accession number J01415.2 in GenBank ). Each multi-alignment refers to the nuclear region (from Chromosome start to Chromosome end), as reported in additional file 1. The NumtS sequences produced in our validation experiments are named with a code defined by the NumtS code and the haplogroup of the sample. The corresponding mtDNA regions are coded as mt, followed by the haplogroup code. [file 1471-2164-9-267-S5.pdf]

NumtS 2

```
                                            20                                              40                                              60
n2_H2b     AAAAGGAACT TGGCAAATTT TTACCCCGCC TGTTTACCGA AAACATCACC TCTAGCACTG TCAGTATTAG 70
n2_hg18    AAAAGGAACT TGGCAAATTT TTACCCCGCC TGTTTACCGA AAACATCACC TCTAGCACTG TCAGTATTAG 70
mt_H2b     AAAAGGAACT CGGCAAA-TC TTACCCCGCC TGTTTACCAA AAACATCACC TCTAGCATCA CCAGTATTAG 69
Consensus  AAAAGGAACT TGGCAAATTT TTACCCCGCC TGTTTACCGA AAACATCACC TCTAGCACTG TCAGTATTAG
Conservation 100% 0%

                    80                                             100                                             120                                             140
n2_H2b     AGGCCCTGCC TGCCCAGTGA CATGTGTTTA ACGTCCACGG TACCCTGACT GTGCAAAGGT AGCATAATCA 140
n2_hg18    AGGCCCTGCC TGCCCAGTGA CATGTGTTTA ACGTCCACGG TACCCTGACT GTGCAAAGGT AGCATAATCA 140
mt_H2b     AGGCACCGCC TGCCCAGTGA CACATGTTTA ACGGCCGCGG TACCCTAACC GTGCAAAGGT AGCATAATCA 139
Consensus  AGGCCCTGCC TGCCCAGTGA CATGTGTTTA ACGTCCACGG TACCCTGACT GTGCAAAGGT AGCATAATCA
Conservation 100% 0%

                                            160                                             180                                             200
n2_H2b     CTTGTTCCTT AAATAGGGAC TTGTATGAAT GGCCCCATGA GGGTTCAGCT GTCTTTTACT TTTAACCAGT 210
n2_hg18    CTTGTTCCTT AAATAGGGAC TTGTATGAAT GGCCCCATGA GGGTTCAGCT GTCTTTTACT TTTAACCAGT 210
mt_H2b     CTTGTTCCTT AAATAGGGAC CTGTATGAAT GGCTCCACGA GGGTTCAGCT GTCTCTTACT TTTAACCAGT 209
Consensus  CTTGTTCCTT AAATAGGGAC TTGTATGAAT GGCCCCATGA GGGTTCAGCT GTCTTTTACT TTTAACCAGT
Conservation 100% 0%

n2_H2b     G 211
n2_hg18    G 211
mt_H2b     G 210
Consensus  G
Conservation 100% 0%
```

NumtS 12

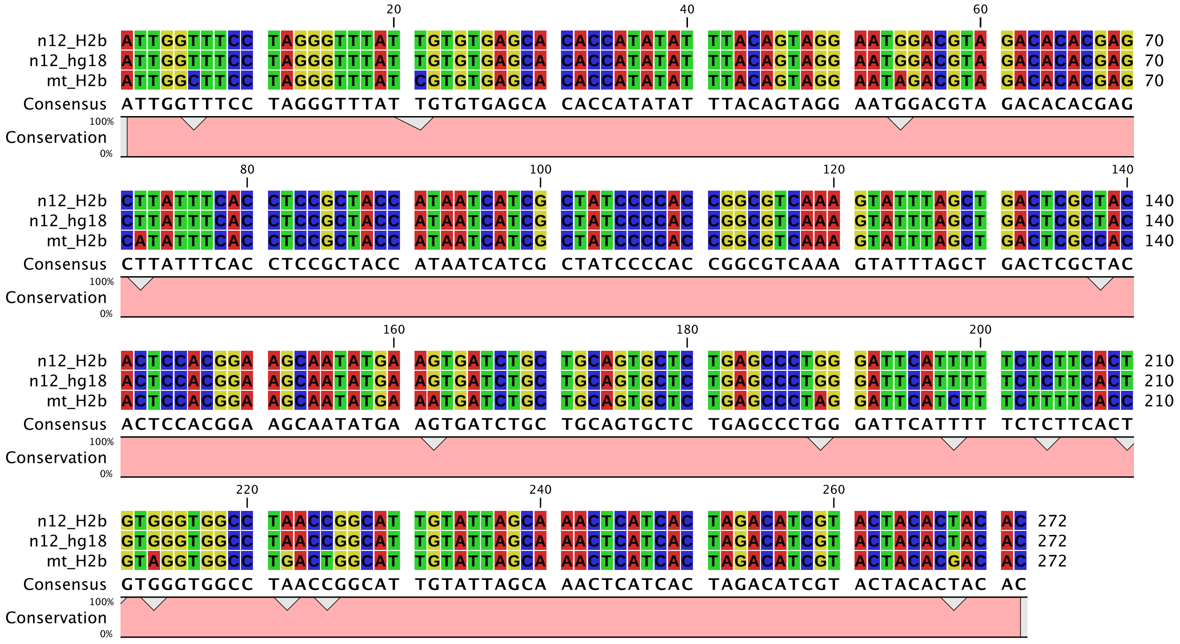

n12_H2b ATTGGTTTCC TAGGGTTTAT TGTGTGAGCA CACCATATAT TTACAGTAGG AATGGACGTA GACACACGAG 70
n12_hg18 ATTGGTTTCC TAGGGTTTAT TGTGTGAGCA CACCATATAT TTACAGTAGG AATGGACGTA GACACACGAG 70
mt_H2b ATTGGCTTCC TAGGGTTTAT CGTGTGAGCA CACCATATAT TTACAGTAGG AATAGACGTA GACACACGAG 70
Consensus ATTGGTTTCC TAGGGTTTAT TGTGTGAGCA CACCATATAT TTACAGTAGG AATGGACGTA GACACACGAG
Conservation
n12_H2b CTTATTTCAC CTCCGCTACC ATAATCATCG CTATCCCCAC CGGCGTCAAA GTATTTAGCT GACTCGCTAC 140
n12_hg18 CTTATTTCAC CTCCGCTACC ATAATCATCG CTATCCCCAC CGGCGTCAAA GTATTTAGCT GACTCGCTAC 140
mt_H2b CATATTTCAC CTCCGCTACC ATAATCATCG CTATCCCCAC CGGCGTCAAA GTATTTAGCT GACTCGCCAC 140
Consensus CTTATTTCAC CTCCGCTACC ATAATCATCG CTATCCCCAC CGGCGTCAAA GTATTTAGCT GACTCGCTAC
Conservation
n12_H2b ACTCCACGGA AGCAATATGA AGTGATCTGC TGCAGTGCTC TGAGCCCTGG GATTCATTTT TCTCTTCACT 210
n12_hg18 ACTCCACGGA AGCAATATGA AGTGATCTGC TGCAGTGCTC TGAGCCCTGG GATTCATTTT TCTCTTCACT 210
mt_H2b ACTCCACGGA AGCAATATGA AATGATCTGC TGCAGTGCTC TGAGCCCTAG GATTCATCTT TCTTTTCACC 210
Consensus ACTCCACGGA AGCAATATGA AGTGATCTGC TGCAGTGCTC TGAGCCCTGG GATTCATTTT TCTCTTCACT
Conservation
n12_H2b GTGGGTGGCC TAACCGGCAT TGTATTAGCA AACTCATCAC TAGACATCGT ACTACACTAC AC 272
n12_hg18 GTGGGTGGCC TAACCGGCAT TGTATTAGCA AACTCATCAC TAGACATCGT ACTACACTAC AC 272
mt_H2b GTAGGTGGCC TGACTGGCAT TGTATTAGCA AACTCATCAC TAGACATCGT ACTACACGAC AC 272
Consensus GTGGGTGGCC TAACCGGCAT TGTATTAGCA AACTCATCAC TAGACATCGT ACTACACTAC AC
Conservation

NumtS 13

```
                                 20                    40                    60
n13_H2b    CCTTAGCAGG AAACTACTCC CACCCAGGAG CCTCTGTAGA CCTGACCATT TTCTCCCTGC ATCTAGCAGG  70
n13_hg18   CCTTAGCAGG AAACTACTCC CACCCAGGAG CCTCTGTAGA CCTGACCATT TTCTCCCTGC ATCTAGCAGG  70
mt_H2b     CCTTAGCAGG GAACTACTCC CACCCTGGAG CCTCCGTAGA CCTAACCATC TTCTCCTTAC ACCTAGCAGG  70
Consensus  CCTTAGCAGG AAACTACTCC CACCCAGGAG CCTCTGTAGA CCTGACCATT TTCTCCCTGC ATCTAGCAGG
Conservation 100% 0%

                      80                   100                   120                   140
n13_H2b    TATCTCCTCC ATTCTAGGGG CTATTAACTT CATTGTGACA ATTATTAACA T-AAACCCCC TGCCATATCT  139
n13_hg18   TATCTCCTCC ATTCTAGGGG CTATTAACTT CATTGTGACA ATTATTAACA T-AAACCCCC TGCCATATCT  139
mt_H2b     TGTCTCCTCT ATCTTAGGGG CCATCAATTT CATCACAACA ATTATCAATA TAAAACCCCC TGCCATAACC  140
Consensus  TATCTCCTCC ATTCTAGGGG CTATTAACTT CATTGTGACA ATTATTAACA T-AAACCCCC TGCCATATCT
Conservation 100% 0%

                                160                   180                   200
n13_H2b    CAATACCAAA CACCCCTCTT TGTCTGATCC GTCCTAATCA CAGCAGTCCT ACTCCTCCTC TCCCTTCCAG  209
n13_hg18   CAATACCAAA CACCCCTCTT TGTCTGATCC GTCCTAATCA CAGCAGTCCT ACTCCTCCTC TCCCTTCCAG  209
mt_H2b     CAATACCAAA CGCCCCTCTT CGTCTGATCC GTCCTAATCA CAGCAGTCCT ACTTCTCCTA TCTCTCCCAG  210
Consensus  CAATACCAAA CACCCCTCTT TGTCTGATCC GTCCTAATCA CAGCAGTCCT ACTCCTCCTC TCCCTTCCAG
Conservation 100% 0%

                     220                   240                   260                   280
n13_H2b    TCCTGGCCGC TGGCATTACT ATATTATTAA CAGACCACAA CCTTGATACC ACCTTCTTTG ATCCAGTCGG  279
n13_hg18   TCCTGGCCGC TGGCATTACT ATATTATTAA CAGACCACAA CCTTGATACC ACCTTCTTTG ATCCAGTCGG  279
mt_H2b     TCCTAGCTGC TGGCATCACT ATACTACTAA CAGACCGCAA CCTCAACACC ACCTTCTTCG ACCCCGCCGG  280
Consensus  TCCTGGCCGC TGGCATTACT ATATTATTAA CAGACCACAA CCTTGATACC ACCTTCTTTG ATCCAGTCGG
Conservation 100% 0%

                                300                   320                   340
n13_H2b    AGGGGGAGAC CCTATCCTGT ACCAACACCT ATTTTGATTC TTCGGTCACC CCAAAGTCTA TATTCTCATC  349
n13_hg18   AGGGGGAGAC CCTATCCTGT ACCAACACCT ATTTTGATTC TTCGGTCACC CCAAAGTCTA TATTCTCATC  349
mt_H2b     AGGAGGAGAC CCCATTCTAT ACCAACACCT ATTCTGATTT TTCGGTCACC CTGAAGTTTA TATTCTTATC  350
Consensus  AGGGGGAGAC CCTATCCTGT ACCAACACCT ATTTTGATTC TTCGGTCACC CCAAAGTCTA TATTCTCATC
Conservation 100% 0%

                     360                   380                   400                   420
n13_H2b    CTACCGGGCT TCGGGATAAT TTCCCATATC GTAACATACT ATTCTGGAAA AAAAGAACCA TTCGGGTATA  419
n13_hg18   CTACCGGGCT TCGGGATAAT TTCCCATATC GTAACATACT ATTCTGGAAA AAAAGAACCA TTCGGGTATA  419
mt_H2b     CTACCAGGCT TCGGAATAAT CTCCCATATT GTAACTTACT ACTCCGGAAA AAAAGAACCA TTTGGATACA  420
Consensus  CTACCGGGCT TCGGGATAAT TTCCCATATC GTAACATACT ATTCTGGAAA AAAAGAACCA TTCGGGTATA
Conservation 100% 0%

                                440                   460                   480
n13_H2b    TGGGCATAGT CTGAGCTATG AAATCAATTG GTTTCCTAGG ATTTATTSTA TGAGCTCATC ATATATTCAC  489
n13_hg18   TGGGCATAGT CTGAGCTATG AAATCAATTG GTTTCCTAGG ATTTATTGTA TGAGCTCATC ATATATTCAC  489
mt_H2b     TAGGTATGGT CTGAGCTATG ATATCAATTG GCTTCCTAGG GTTTATCGTG TGAGCACACC ATATATTTAC  490
Consensus  TGGGCATAGT CTGAGCTATG AAATCAATTG GTTTCCTAGG ATTTATTGTA TGAGCTCATC ATATATTCAC
Conservation 100% 0%

                     500                   520                   540                   560
n13_H2b    AGTAGGGATA GACGTGGATA CACGAGCCTA CTTCACCTCC GCTACTATAA TTATCGCCAT TTCCACTGGC  559
n13_hg18   AGTAGGGATA GACGTGGATA CACGAGCCTA CTTCACCTCC GCTACTATAA TTATCGCCAT TTCCACTGGC  559
mt_H2b     AGTAGGAATA GACGTAGACA CACGAGCATA TTTCACCTCC GCTACCATAA TCATCGCTAT CCCCACCGGC  560
Consensus  AGTAGGGATA GACGTGGATA CACGAGCCTA CTTCACCTCC GCTACTATAA TTATCGCCAT TTCCACTGGC
Conservation 100% 0%

                                580                   600                   620
n13_H2b    GTCAAAGTAT TTAGYTGACT TGCTACGCTT CACAGAAGTA ACATCAAATG ATCTGCCGCG GTACTCTGAG  629
n13_hg18   GTCAAAGTAT TTAGTTGACT TGCTACGCTT CACAGAAGTA ACATCAAATG ATCTGCCGCG GTACTCTGAG  629
mt_H2b     GTCAAAGTAT TTAGCTGACT CGCCACACTC CACGGAAGCA ATATGAAATG ATCTGCTGCA GTGCTCTGAG  630
Consensus  GTCAAAGTAT TTAGNTGACT TGCTACGCTT CACAGAAGTA ACATCAAATG ATCTGCCGCG GTACTCTGAG
Conservation 100% 0%

                     640                   660                   680                   700
n13_H2b    CCGTAGGGTT CATCTTCCTC TTCACTGTGG GTAGTCTAAC CGGCATTGTA CTAGCAAACT CATCATTAGA  699
n13_hg18   CCGTAGGGTT CATCTTCCTC TTCACTGTGG GTAGTCTAAC CGGCATTGTA CTAGCAAACT CATCATTAGA  699
mt_H2b     CCCTAGGATT CATCTTTCTT TTCACCGTAG GTGGCCTGAC TGGCATTGTA TTAGCAAACT CATCACTAGA  700
Consensus  CCGTAGGGTT CATCTTCCTC TTCACTGTGG GTAGTCTAAC CGGCATTGTA CTAGCAAACT CATCATTAGA
Conservation 100% 0%
```

NumtS 28

```
                                           20                    40                    60
n28_H2b       GAAACTGTTT AGACGGGCTC ACATCACCCC ATAAACAAAT AGGTTTGGTC CTAGCCTTTC TATTAGCTCT  70
n28_hg18      GAAACTGTTT AGACGGGCTC ACATCACCCC ATAAACAAAT AGGTTTGGTC CTAGCCTTTC TATTAGCTCT  70
mt_H2b        GAAAATGTTT AGACGGGCTC ACATCACCCC ATAAACAAAT AGGTTTGGTC CTAGCCTTTC TATTAGCTCT  70
Consensus     GAAACTGTTT AGACGGGCTC ACATCACCCC ATAAACAAAT AGGTTTGGTC CTAGCCTTTC TATTAGCTCT
        100%
Conservation
          0%

                        80                    100                   120
n28_H2b       TAGTAAGATT ACACATGCAA GCATCCCCAT TCCAGTGAGT TCACCCTCTA AATCACCACG AT  132
n28_hg18      TAGTAAGATT ACACATGCAA GCATCCCCAT TCCAGTGAGT TCACCCTCTA AATCACCACG AT  132
mt_H2b        TAGTAAGATT ACACATGCAA GCATCCCCGT TCCAGTGAGT TCACCCTCTA AATCACCACG AT  132
Consensus     TAGTAAGATT ACACATGCAA GCATCCCCAT TCCAGTGAGT TCACCCTCTA AATCACCACG AT
        100%
Conservation
          0%
```

NumtS 38

```
                                    20                                      40                                      60
                                     |                                       |                                       |
n38_H2b    CCAGCCACCG CGGTCACACG ATTAACCCAA GTCAATAGAA ACCGGCGTAA AGAGTGTTTT AGATTAATTC  70
n38_hg18   CCAGCCACCG CGGTCACACG ATTAACCCAA GTCAATAGAA ACCGGCGTAA AGAGTGTTTT AGATTAATTC  70
mt_H2b     CCAGCCACCG CGGTCACACG ATTAACCCAA GTCAATAGAA GCCGGCGTAA AGAGTGTTTT AGATCACCCC  70
Consensus  CCAGCCACCG CGGTCACACG ATTAACCCAA GTCAATAGAA ACCGGCGTAA AGAGTGTTTT AGATTAATTC
```

Conservation

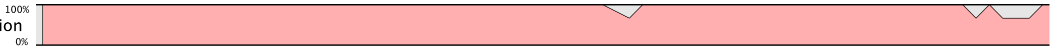


```
                    80                                     100                                     120                                     140
                     |                                       |                                       |                                       |
n38_H2b    C-CCTCAATA AAGCTAAAAT TCACCTGAGT TGTAAAAAAC TCCAGCTGAT ACAAAATAAA CTACGAAAGT  139
n38_hg18   C-CCTCAATA AAGCTAAAAT TCACCTGAGT TGTAAAAAAC TCCAGCTGAT ACAAAATAAA CTACGAAAGT  139
mt_H2b     CTCCCCAATA AAGCTAAAAC TCACCTGAGT TGTAAAAAAC TCCAGTTGAC ACAAAATAGA CTACGAAAGT  140
Consensus  C-CCTCAATA AAGCTAAAAT TCACCTGAGT TGTAAAAAAC TCCAGCTGAT ACAAAATAAA CTACGAAAGT
```

Conservation

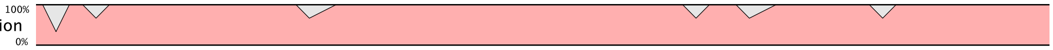


```
n38_H2b    GGCTTTAACG CATCTGAAC  158
n38_hg18   GGCTTTAACG CATCTGAAC  158
mt_H2b     GGCTTTAACA TATCTGAAC  159
Consensus  GGCTTTAACG CATCTGAAC
```

Conservation

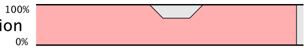

NumtS 63

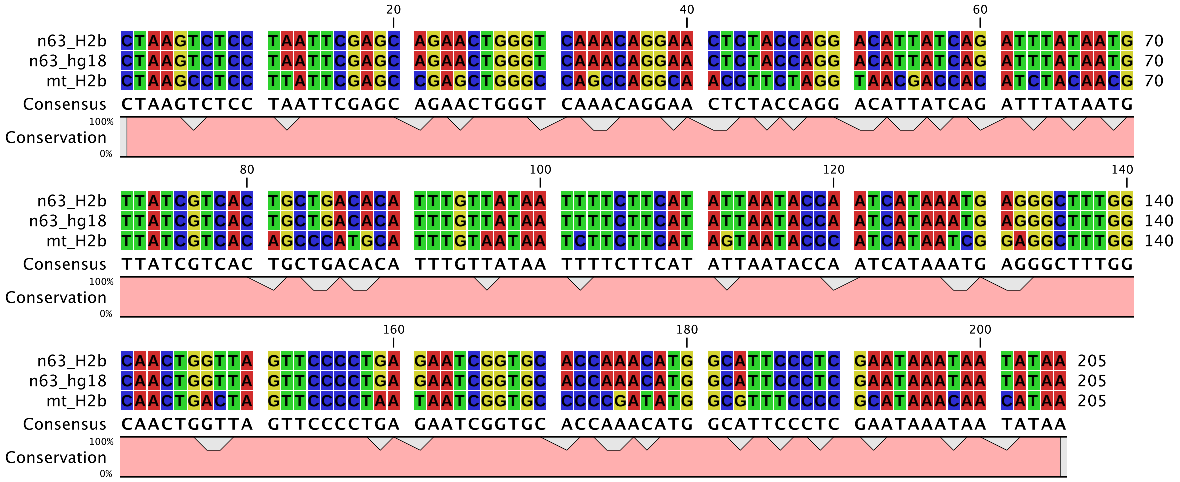
20 40 60
n63_H2b CTAAGTCTCC TAATTCGAGC AGAACTGGGT CAAACAGGAA CTCTACCAGG ACATTATCAG ATTTATAATG 70
n63_hg18 CTAAGTCTCC TAATTCGAGC AGAACTGGGT CAAACAGGAA CTCTACCAGG ACATTATCAG ATTTATAATG 70
mt_H2b CTAAGCCTCC TTATTCGAGC CGAGCTGGGC CAGCCAGGCA ACCTTCTAGG TAACGACCAC ATCTACAACG 70
Consensus CTAAGTCTCC TAATTCGAGC AGAACTGGGT CAAACAGGAA CTCTACCAGG ACATTATCAG ATTTATAATG
Conservation 100% 0%
80 100 120 140
n63_H2b TTATCGTCAC TGCTGACACA TTTGTTATAA TTTTCTTCAT ATTAATACCA ATCATAAATG AGGGCTTTGG 140
n63_hg18 TTATCGTCAC TGCTGACACA TTTGTTATAA TTTTCTTCAT ATTAATACCA ATCATAAATG AGGGCTTTGG 140
mt_H2b TTATCGTCAC AGCCCATGCA TTTGTAATAA TCTTCTTCAT AGTAATACCC ATCATAATCG GAGGCTTTGG 140
Consensus TTATCGTCAC TGCTGACACA TTTGTTATAA TTTTCTTCAT ATTAATACCA ATCATAAATG AGGGCTTTGG
Conservation 100% 0%
160 180 200
n63_H2b CAACTGGTTA GTTCCCCTGA GAATCGGTGC ACCAAACATG GCATTCCCTC GAATAAATAA TATAA 205
n63_hg18 CAACTGGTTA GTTCCCCTGA GAATCGGTGC ACCAAACATG GCATTCCCTC GAATAAATAA TATAA 205
mt_H2b CAACTGACTA GTTCCCCTAA TAATCGGTGC CCCCGATATG GCGTTTCCCC GCATAAACAA CATAA 205
Consensus CAACTGGTTA GTTCCCCTGA GAATCGGTGC ACCAAACATG GCATTCCCTC GAATAAATAA TATAA
Conservation 100% 0%

NumtS 72

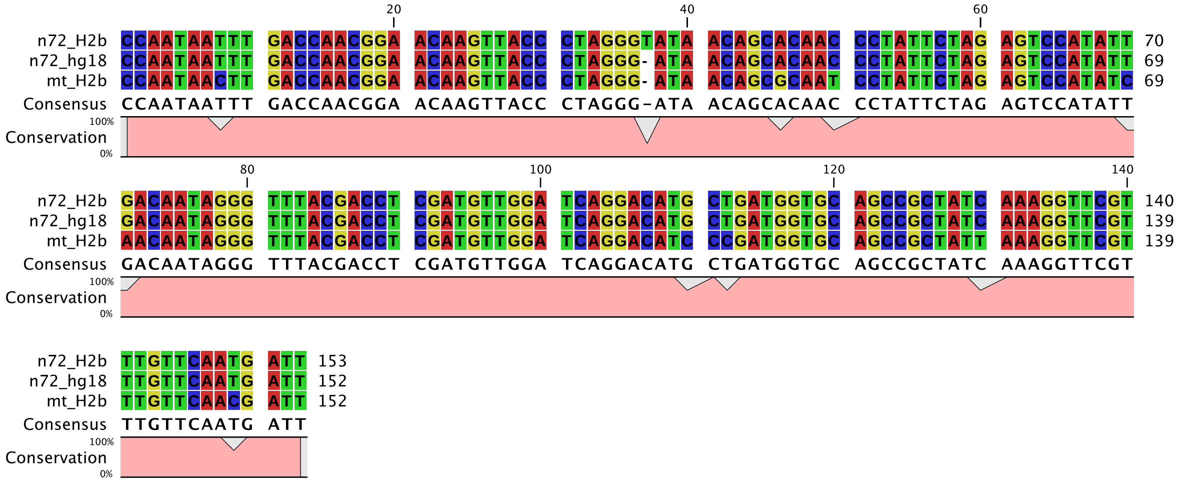

20
40
60
n72_H2b CCAATAATTT GACCAACGGA ACAAGTTACC CTAGGGTATA ACAGCACAAC CCTATTCTAG AGTCCATATT 70
n72_hg18 CCAATAATTT GACCAACGGA ACAAGTTACC CTAGGG- ATA ACAGCACAAC CCTATTCTAG AGTCCATATT 69
mt_H2b CCAATAACTT GACCAACGGA ACAAGTTACC CTAGGG- ATA ACAGCGCAAT CCTATTCTAG AGTCCATATC 69
Consensus CCAATAATTT GACCAACGGA ACAAGTTACC CTAGGG-ATA ACAGCACAAC CCTATTCTAG AGTCCATATT
Conservation
100%
0%
80
100
120
140
n72_H2b GACAATAGGG TTTACGACCT CGATGTTGGA TCAGGACATG CTGATGGTGC AGCCGCTATC AAAGGTTCGT 140
n72_hg18 GACAATAGGG TTTACGACCT CGATGTTGGA TCAGGACATG CTGATGGTGC AGCCGCTATC AAAGGTTCGT 139
mt_H2b AACAATAGGG TTTACGACCT CGATGTTGGA TCAGGACATC CCGATGGTGC AGCCGCTATT AAAGGTTCGT 139
Consensus GACAATAGGG TTTACGACCT CGATGTTGGA TCAGGACATG CTGATGGTGC AGCCGCTATC AAAGGTTCGT
Conservation
100%
0%
n72_H2b TTGTTCAATG ATT 153
n72_hg18 TTGTTCAATG ATT 152
mt_H2b TTGTTCAACG ATT 152
Consensus TTGTTCAATG ATT
Conservation
100%
0%

NumtS 73

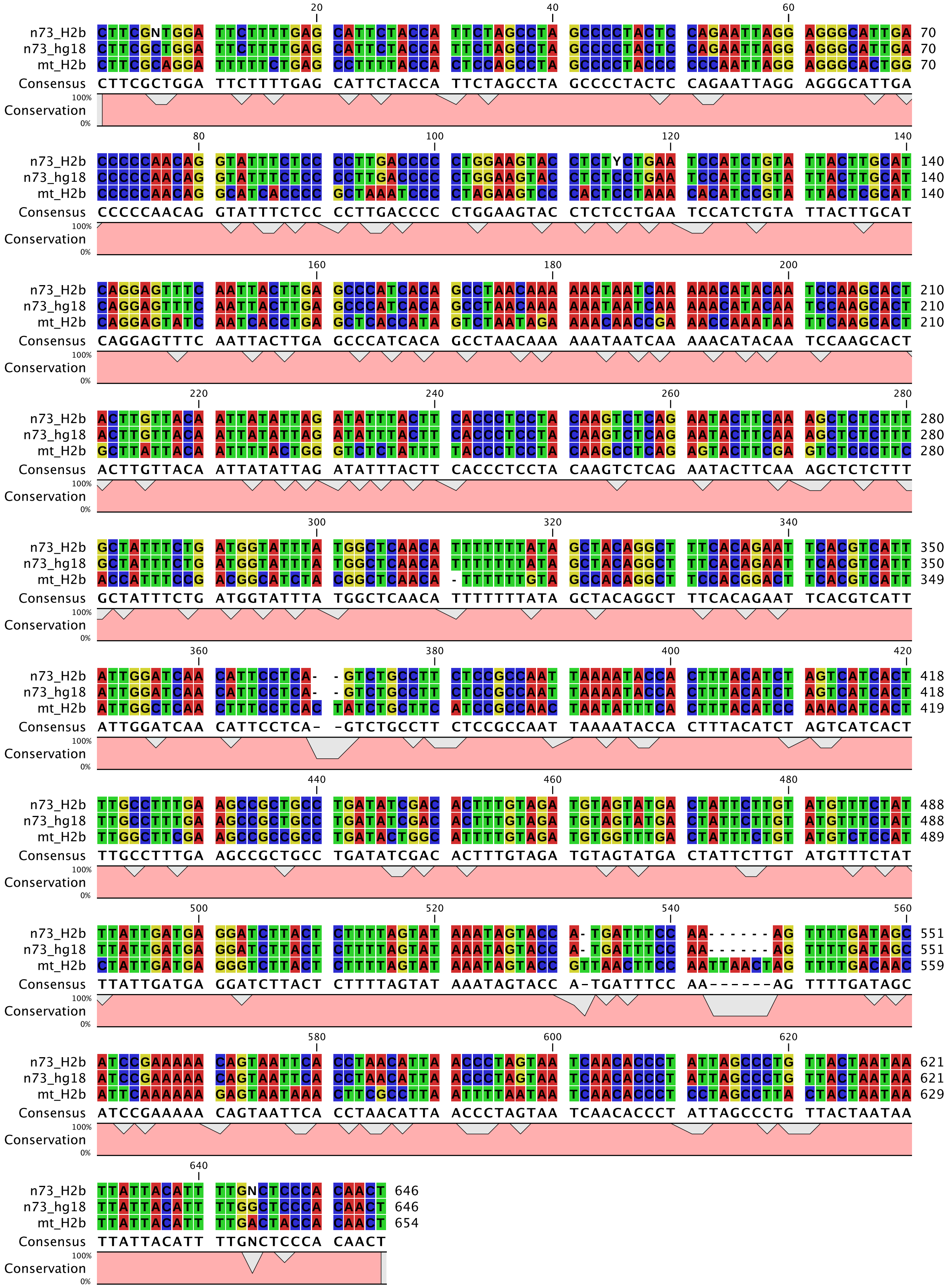

n73_H2b CTTCGNTGGA TTCTTTTGAG CATTCTACCA TTCTAGCCTA GCCCCTACTC CAGAATTAGG AGGGCATTGA 70
n73_hg18 CTTCGCTGGA TTCTTTTGAG CATTCTACCA TTCTAGCCTA GCCCCTACTC CAGAATTAGG AGGGCATTGA 70
mt_H2b CTTCGCAGGA TTTTTCTGAG CCTTTTACCA CTCCAGCCTA GCCCCTACCC CCCAATTAGG AGGGCACTGG 70
Consensus CTTCGCTGGA TTCTTTTGAG CATTCTACCA TTCTAGCCTA GCCCCTACTC CAGAATTAGG AGGGCATTGA
Conservation
n73_H2b CCCCCAACAG GTATTTCTCC CCTTGACCCC CTGGAAGTAC CTCTYCTGAA TCCATCTGTA TTACTTGCAT 140
n73_hg18 CCCCCAACAG GTATTTCTCC CCTTGACCCC CTGGAAGTAC CTCTCCTGAA TCCATCTGTA TTACTTGCAT 140
mt_H2b CCCCCAACAG GCATCACCCC GCTAAATCCC CTAGAAGTCC CACTCCTAAA CACATCCGTA TTACTCGCAT 140
Consensus CCCCCAACAG GTATTTCTCC CCTTGACCCC CTGGAAGTAC CTCTCCTGAA TCCATCTGTA TTACTTGCAT
Conservation
n73_H2b CAGGAGTTTC AATTACTTGA GCCCATCACA GCCTAACAAA AAATAATCAA AAACATACAA TCCAAGCACT 210
n73_hg18 CAGGAGTTTC AATTACTTGA GCCCATCACA GCCTAACAAA AAATAATCAA AAACATACAA TCCAAGCACT 210
mt_H2b CAGGAGTATC AATCACCTGA GCTCACCATA GTCTAATAGA AAACAACCGA AACCAAATAA TTCAAGCACT 210
Consensus CAGGAGTTTC AATTACTTGA GCCCATCACA GCCTAACAAA AAATAATCAA AAACATACAA TCCAAGCACT
Conservation
n73_H2b ACTTGTTACA ATTATATTAG ATATTTACTT CACCCTCCTA CAAGTCTCAG AATACTTCAA AGCTCTCTTT 280
n73_hg18 ACTTGTTACA ATTATATTAG ATATTTACTT CACCCTCCTA CAAGTCTCAG AATACTTCAA AGCTCTCTTT 280
mt_H2b GCTTATTACA ATTTTACTGG GTCTCTATTT TACCCTCCTA CAAGCCTCAG AGTACTTCGA GTCTCCCTTC 280
Consensus ACTTGTTACA ATTATATTAG ATATTTACTT CACCCTCCTA CAAGTCTCAG AATACTTCAA AGCTCTCTTT
Conservation
n73_H2b GCTATTTCTG ATGGTATTTA TGGCTCAACA TTTTTTTATA GCTACAGGCT TTCACAGAAT TCACGTCATT 350
n73_hg18 GCTATTTCTG ATGGTATTTA TGGCTCAACA TTTTTTTATA GCTACAGGCT TTCACAGAAT TCACGTCATT 350
mt_H2b ACCATTTCCG ACGGCATCTA CGGCTCAACA -TTTTTTGTA GCCACAGGCT TCCACGGACT TCACGTCATT 349
Consensus GCTATTTCTG ATGGTATTTA TGGCTCAACA TTTTTTTATA GCTACAGGCT TTCACAGAAT TCACGTCATT
Conservation
n73_H2b ATTGGATCAA CATTCCTCA- -GTCTGCCTT CTCCGCCAAT TAAAATACCA CTTTACATCT AGTCATCACT 418
n73_hg18 ATTGGATCAA CATTCCTCA- -GTCTGCCTT CTCCGCCAAT TAAAATACCA CTTTACATCT AGTCATCACT 418
mt_H2b ATTGGCTCAA CTTTCCTCAC TATCTGCTTC ATCCGCCAAC TAATATTTCA CTTTACATCC AAACATCACT 419
Consensus ATTGGATCAA CATTCCTCA- -GTCTGCCTT CTCCGCCAAT TAAAATACCA CTTTACATCT AGTCATCACT
Conservation
n73_H2b TTGCCTTTGA AGCCGCTGCC TGATATCGAC ACTTTGTAGA TGTAGTATGA CTATTCTTGT ATGTTTCTAT 488
n73_hg18 TTGCCTTTGA AGCCGCTGCC TGATATCGAC ACTTTGTAGA TGTAGTATGA CTATTCTTGT ATGTTTCTAT 488
mt_H2b TTGGCTTCGA AGCCGCCGCC TGATACTGGC ATTTTGTAGA TGTGGTTTGA CTATTTCTGT ATGTCTCCAT 489
Consensus TTGCCTTTGA AGCCGCTGCC TGATATCGAC ACTTTGTAGA TGTAGTATGA CTATTCTTGT ATGTTTCTAT
Conservation
n73_H2b TTATTGATGA GGATCTTACT CTTTTAGTAT AAATAGTACC A-TGATTTCC AA------AG TTTTGATAGC 551
n73_hg18 TTATTGATGA GGATCTTACT CTTTTAGTAT AAATAGTACC A-TGATTTCC AA------AG TTTTGATAGC 551
mt_H2b CTATTGATGA GGGTCTTACT CTTTTAGTAT AAATAGTACC GTTAACTTCC AATTAACTAG TTTTGACAAC 559
Consensus TTATTGATGA GGATCTTACT CTTTTAGTAT AAATAGTACC A-TGATTTCC AA------AG TTTTGATAGC
Conservation
n73_H2b ATCCGAAAAA CAGTAATTCA CCTAACATTA ACCCTAGTAA TCAACACCCT ATTAGCCCTG TTACTAATAA 621
n73_hg18 ATCCGAAAAA CAGTAATTCA CCTAACATTA ACCCTAGTAA TCAACACCCT ATTAGCCCTG TTACTAATAA 621
mt_H2b ATTCAAAAAA GAGTAATAAA CTTCGCCTTA ATTTTAATAA TCAACACCCT CCTAGCCTTA CTACTAATAA 629
Consensus ATCCGAAAAA CAGTAATTCA CCTAACATTA ACCCTAGTAA TCAACACCCT ATTAGCCCTG TTACTAATAA
Conservation
n73_H2b TTATTACATT TTGNCTCCCA CAACT 646
n73_hg18 TTATTACATT TTGGCTCCCA CAACT 646
mt_H2b TTATTACATT TTGACTACCA CAACT 654
Consensus TTATTACATT TTGNCTCCCA CAACT
Conservation

NumtS 75

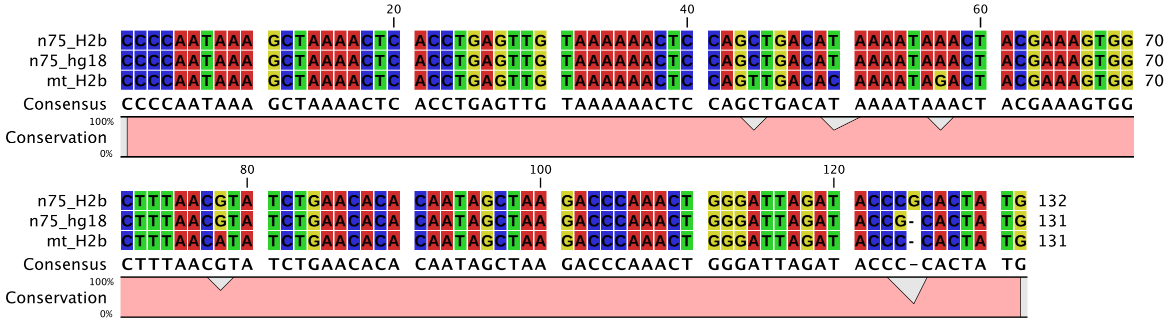
20 40 60
n75_H2b CCCCAATAAA GCTAAAACTC ACCTGAGTTG TAAAAAACTC CAGCTGACAT AAAATAAACT ACGAAAGTGG 70
n75_hg18 CCCCAATAAA GCTAAAACTC ACCTGAGTTG TAAAAAACTC CAGCTGACAT AAAATAAACT ACGAAAGTGG 70
mt_H2b CCCCAATAAA GCTAAAACTC ACCTGAGTTG TAAAAAACTC CAGTTGACAC AAAATAGACT ACGAAAGTGG 70
Consensus CCCCAATAAA GCTAAAACTC ACCTGAGTTG TAAAAAACTC CAGCTGACAT AAAATAAACT ACGAAAGTGG
Conservation
100%
0%
80 100 120
n75_H2b CTTTAACGTA TCTGAACACA CAATAGCTAA GACCCAAACT GGGATTAGAT ACCCGCACTA TG 132
n75_hg18 CTTTAACGTA TCTGAACACA CAATAGCTAA GACCCAAACT GGGATTAGAT ACCG-CACTA TG 131
mt_H2b CTTTAACATA TCTGAACACA CAATAGCTAA GACCCAAACT GGGATTAGAT ACCC-CACTA TG 131
Consensus CTTTAACGTA TCTGAACACA CAATAGCTAA GACCCAAACT GGGATTAGAT ACCC-CACTA TG
Conservation
100%
0%

NumtS 77

```
                                            20                                      40                                      60
n77_H2b    CTAAAAAATC  CCAAACATAC  AACTGAGCTC  CTTACACTCA  ATTGGACTAA  TCTATTACCT  TATAGAAGAA  70
n77_hg18   CTAAAAAATC  CCAAACATAC  AACTGAGCTC  CTTACACTCA  ATTGGACTAA  TCTATTACCT  TATAGAAGAA  70
mt_H2b     -TAAAAAATC  CCAAACATAT  AACTGAACTC  CTCACACCCA  ATTGGACCAA  TCTATCACCC  TATAGAAGAA  69
Consensus  CTAAAAAATC  CCAAACATAC  AACTGAGCTC  CTTACACTCA  ATTGGACTAA  TCTATTACCT  TATAGAAGAA
      100%
Conservation
        0%
                              80                                     100                                     120                                     140
n77_H2b    CTAATGTTAG  TATAAGTAAC  ATGAAAACAT  TCTCCTCCTC  ATAAGCCTAC  ATCAGACCAA  AATATTAAAC  140
n77_hg18   CTAATGTTAG  TATAAGTAAC  ATGAAAACAT  TCTCCTCCTC  ATAAGCCTAC  ATCAGACCAA  AATATTAAAC  140
mt_H2b     CTAATGTTAG  TATAAGTAAC  ATGAAAACAT  TCTCCTCCGC  ATAAGCCTGC  GTCAGATTAA  AACACTGAAC  139
Consensus  CTAATGTTAG  TATAAGTAAC  ATGAAAACAT  TCTCCTCCTC  ATAAGCCTAC  ATCAGACCAA  AATATTAAAC
      100%
Conservation
        0%
                                           160                                     180                                     200
n77_H2b    TGACAATTAA  CAGCCCAATA  TCTACAATCA  ACCAACAAGC  CATTATTACC  CCCACTGTTA  ACCCAACACA  210
n77_hg18   TGACAATTAA  CAGCCCAATA  TCTACAATCA  ACCAACAAGC  CATTATTACC  CCCACTGTTA  ACCCAACACA  210
mt_H2b     TGACAATTAA  CAGCCCAATA  TCTACAATCA  ACCAACAAGT  CATTATTACC  CTCACTGTCA  ACCCAACACA  209
Consensus  TGACAATTAA  CAGCCCAATA  TCTACAATCA  ACCAACAAGC  CATTATTACC  CCCACTGTTA  ACCCAACACA
      100%
Conservation
        0%
                 220
n77_H2b    GGCATGCCCA  CAAGGAAAGG  TT  232
n77_hg18   GGCATGCCCA  CAAGGAAAGG  TT  232
mt_H2b     GGCATGCTCA  TAAGGAAAGG  TT  231
Consensus  GGCATGCCCA  CAAGGAAAGG  TT
      100%
Conservation
        0%
```

NumtS 82

```
                                        20                              40                              60
n82_H2b    CAACATGGCT TTCTCAACTT TTAAAGGATA ACAGCCATCC GTTGGTCTTA GGCCCCAAAA ATTTTGGTGC  70
n82_hg18   CAACATGGCT TTCTCAACTT TTAAAGGATA ACAGCCATCC GTTGGTCTTA GGCCCCAAAA ATTTTGGTGC  70
mt_H2b     CAACATGGCT TTCTCAACTT TTAAAGGATA ACAGCTATCC ATTGGTCTTA GGCCCCAAAA ATTTTGGTGC  70
Consensus  CAACATGGCT TTCTCAACTT TTAAAGGATA ACAGCCATCC GTTGGTCTTA GGCCCCAAAA ATTTTGGTGC
      100%
Conservation
        0%
                    80                              100                             120                             140
n82_H2b    AACTCCAAAT AAAAGTAATA ACCATGTATG CCACTATAAC CACCCTAGCC CTAACTTCCT TAATTCCCCC  140
n82_hg18   AACTCCAAAT AAAAGTAATA ACCATGTATG CCACTATAAC CACCCTAGCC CTAACTTCCT TAATTCCCCC  140
mt_H2b     AACTCCAAAT AAAAGTAATA ACCATGCACA CTACTATAAC CACCCTAACC CTGACTTCCC TAATTCCCCC  140
Consensus  AACTCCAAAT AAAAGTAATA ACCATGTATG CCACTATAAC CACCCTAGCC CTAACTTCCT TAATTCCCCC
      100%
Conservation
        0%
                                        160
n82_H2b    CATCCTTACC ACCTTCATTA ACCCTAAC  168
n82_hg18   CATCCTTACC ACCTTCATTA ACCCTAAC  168
mt_H2b     CATCCTTACC ACCCTCGTTA ACCCTAAC  168
Consensus  CATCCTTACC ACCTTCATTA ACCCTAAC
      100%
Conservation
        0%
```

NumtS 101

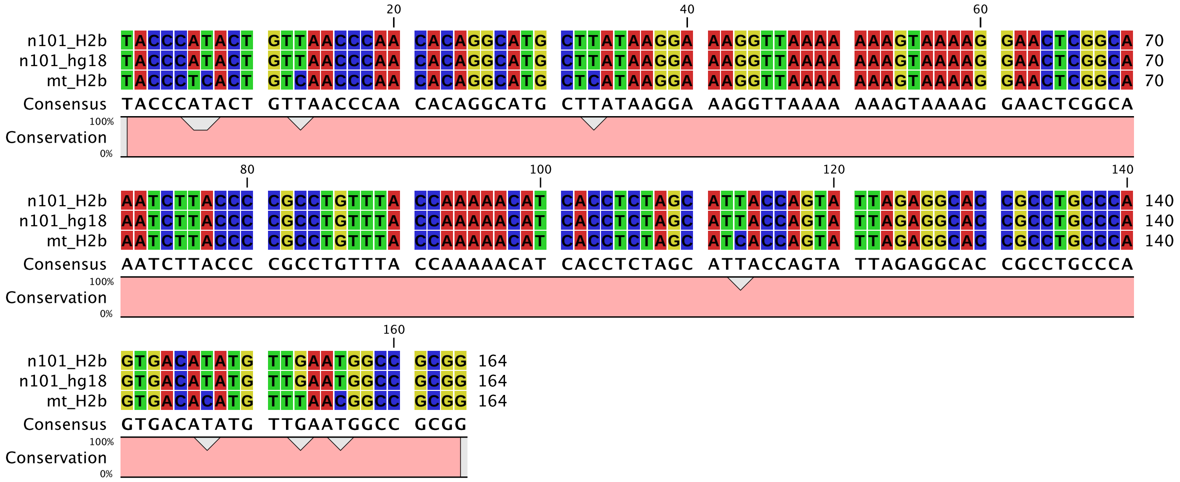
20
40
60
n101_H2b TACCCATACT GTTAACCCAA CACAGGCATG CTTATAAGGA AAGGTTAAAA AAAGTAAAAG GAACTCGGCA 70
n101_hg18 TACCCATACT GTTAACCCAA CACAGGCATG CTTATAAGGA AAGGTTAAAA AAAGTAAAAG GAACTCGGCA 70
mt_H2b TACCCTCACT GTCAACCCAA CACAGGCATG CTCATAAGGA AAGGTTAAAA AAAGTAAAAG GAACTCGGCA 70
Consensus TACCCATACT GTTAACCCAA CACAGGCATG CTTATAAGGA AAGGTTAAAA AAAGTAAAAG GAACTCGGCA
Conservation
100%
0%
80
100
120
140
n101_H2b AATCTTACCC CGCCTGTTTA CCAAAAACAT CACCTCTAGC ATTACCAGTA TTAGAGGCAC CGCCTGCCCA 140
n101_hg18 AATCTTACCC CGCCTGTTTA CCAAAAACAT CACCTCTAGC ATTACCAGTA TTAGAGGCAC CGCCTGCCCA 140
mt_H2b AATCTTACCC CGCCTGTTTA CCAAAAACAT CACCTCTAGC ATCACCAGTA TTAGAGGCAC CGCCTGCCCA 140
Consensus AATCTTACCC CGCCTGTTTA CCAAAAACAT CACCTCTAGC ATTACCAGTA TTAGAGGCAC CGCCTGCCCA
Conservation
100%
0%
160
n101_H2b GTGACATATG TTGAATGGCC GCGG 164
n101_hg18 GTGACATATG TTGAATGGCC GCGG 164
mt_H2b GTGACACATG TTTAACGGCC GCGG 164
Consensus GTGACATATG TTGAATGGCC GCGG
Conservation
100%
0%

NumtS 109

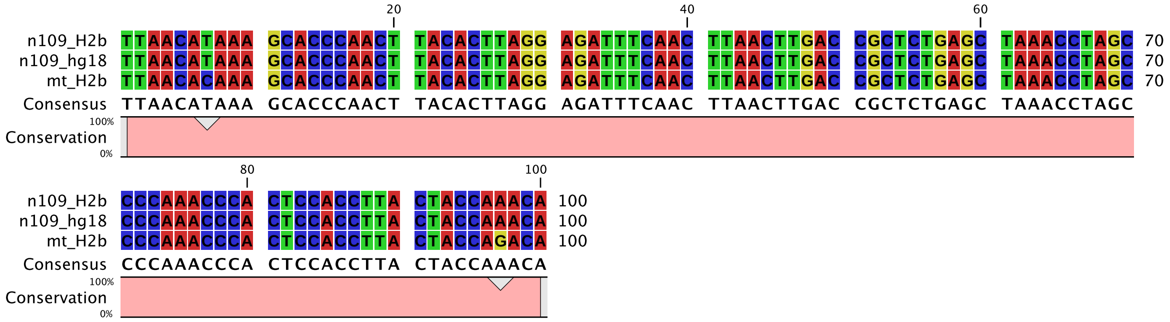

20
40
60
n109_H2b TTAACATAAA GCACCCAACT TACACTTAGG AGATTTCAAC TTAACTTGAC CGCTCTGAGC TAAACCTAGC 70
n109_hg18 TTAACATAAA GCACCCAACT TACACTTAGG AGATTTCAAC TTAACTTGAC CGCTCTGAGC TAAACCTAGC 70
mt_H2b TTAACACAAA GCACCCAACT TACACTTAGG AGATTTCAAC TTAACTTGAC CGCTCTGAGC TAAACCTAGC 70
Consensus TTAACATAAA GCACCCAACT TACACTTAGG AGATTTCAAC TTAACTTGAC CGCTCTGAGC TAAACCTAGC
100%
Conservation
0%
80
100
n109_H2b CCCAAACCCA CTCCACCTTA CTACCAAACA 100
n109_hg18 CCCAAACCCA CTCCACCTTA CTACCAAACA 100
mt_H2b CCCAAACCCA CTCCACCTTA CTACCAGACA 100
Consensus CCCAAACCCA CTCCACCTTA CTACCAAACA
100%
Conservation
0%

NumtS 112

```
                       20                  40                  60
                        |                   |                   |
n112_H2b   CACGGGAGAC AGCAGTGATA AGCCTTTAGC AATAAACGAA AGTTTAACTA AGCTATACTG ACC  63
n112_hg18  CACGGGAGAC AGCAGTGATA AGCCTTTAGC AATAAACGAA AGTTTAACTA AGCTATACTG ACC  63
mt_H2b     CACGGGAAAC AGCAGTGATT AACCTTTAGC AATAAACGAA AGTTTAACTA AGCTATACTA ACC  63
Consensus  CACGGGAGAC AGCAGTGATA AGCCTTTAGC AATAAACGAA AGTTTAACTA AGCTATACTG ACC
```

Conservation

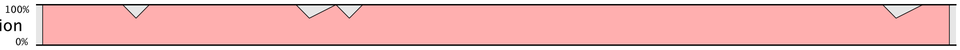

NumtS 115

```
                                   20                            40                            60
n115_H2b    TCATTCCCAC TATTATAGAT ATTATAACCC TACTCAACCT ATACTTTTAT ATATGCCTAA TCTATTCTAC  70
n115_hg18   TCATTCCCAC TATTATAGAT ATTATAACCC TACTCAACCT ATACTTTTAT ATATGCCTAA TCTATTCTAC  70
mt_H2b      TCATCCCCAC CATCATAGCC ACCATCACCC TCCTTAACCT CTACTTCTAC CTACGCCTAA TCTACTCCAC  70
Consensus   TCATTCCCAC TATTATAGAT ATTATAACCC TACTCAACCT ATACTTTTAT ATATGCCTAA TCTATTCTAC
```

Conservation 100% 0%

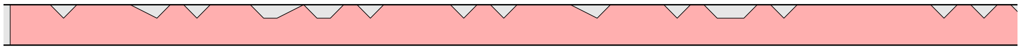

```
                     80                           100                           120                           140
n115_H2b    TTCAGTCACA TTATTCCCCA TATCCAATAA TGTAAAAATA AAGTGACAGT TTGAAAACAC AAAGCCCATA  140
n115_hg18   TTCAGTCACA TTATTCCCCA TATCCAATAA TGTAAAAATA AAGTGACAGT TTGAAAACAC AAAGCCCATA  140
mt_H2b      CTCAATCACA CTACTCCCCA TATCTAACAA CGTAAAAATA AAATGACAGT TTGAACATAC AAAACCCACC  140
Consensus   TTCAGTCACA TTATTCCCCA TATCCAATAA TGTAAAAATA AAGTGACAGT TTGAAAACAC AAAGCCCATA
```

Conservation 100% 0%

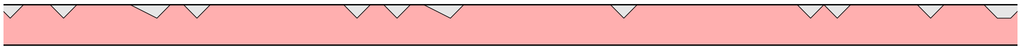

```
                                  160                           180                           200
n115_H2b    CTACTTCTCC CCCGGCTTAC CATCCTCGCC ATCTTCCTCC TACCAATCTC CCTACTTACC CTGCTTATCT  210
n115_hg18   CTACTTCTCC CCCGGCTTAC CATCCTCGCC ATCTTCCTCC TACCAATCTC CCTACTTACC CTGCTTATCT  210
mt_H2b      CCATTCCTCC CCACACTCAT CGCCCTTACC ACGCTACTCC TACCTATCTC CCCTTTTATA CTAATAATCT  210
Consensus   CTACTTCTCC CCCGGCTTAC CATCCTCGCC ATCTTCCTCC TACCAATCTC CCTACTTACC CTGCTTATCT
```

Conservation 100% 0%

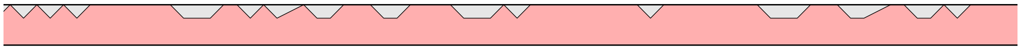

```
                    220                           240                           260                           280
n115_H2b    TCTAGAAATT TAGGTTAAAC A-AGACCAAG AGCCTTCAAA GCCCTTAGTA AGTAGACTAT ACTTAATTTC  279
n115_hg18   TCTAGAAATT TAGGTTAAAC A-AGACCAAG AGCCTTCAAA GCCCTTAGTA AGTAGACTAT ACTTAATTTC  279
mt_H2b      TATAGAAATT TAGGTTAAAT ACAGACCAAG AGCCTTCAAA GCCCTCAGTA AGTTG-CAAT ACTTAATTTC  279
Consensus   TCTAGAAATT TAGGTTAAAC A-AGACCAAG AGCCTTCAAA GCCCTTAGTA AGTAGACTAT ACTTAATTTC
```

Conservation 100% 0%

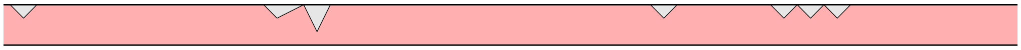

```
                                  300
n115_H2b    TG-AAAACCC AAGGACTGCA AAACTCTACT CTGCA  313
n115_hg18   TG-AAAACCC AAGGACTGCA AAACTCTACT CTGCA  313
mt_H2b      TGTAACAGCT AAGGACTGCA AAACCCCACT CTGCA  314
Consensus   TG-AAAACCC AAGGACTGCA AAACTCTACT CTGCA
```

Conservation 100% 0%

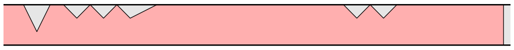

NumtS 133

```
n133_H2b   CACTGTAAAG CTAACCCAGC ATTAACCTTT TAAGTTAAAG ACTAAGAGAA TCATTATCTC TTTACAGTGA 70
n133_hg18  CACTGTAAAG CTAACCCAGC ATTAACCTTT TAAGTTAAAG ACTAAGAGAA TCATTATCTC TTTACAGTGA 70
mt_H2b     CACTGTAAAG CTAACTTAGC ATTAACCTTT TAAGTTAAAG ATTAAGAGAA CCAACACCTC TTTACAGTGA 70
Consensus  CACTGTAAAG CTAACCCAGC ATTAACCTTT TAAGTTAAAG ACTAAGAGAA TCATTATCTC TTTACAGTGA

n133_H2b   AATGCCACAG CTAAATACCA CTGTATGACC TAGCTATCAT CACCCCAAGT ACTCCTCACG TTATTTCTCA 140
n133_hg18  AATGCCACAG CTAAATACCA CTGTATGACC TG-CTATCAT CACCCCAA-T ACTCCTCACG TTATTTCTCA 138
mt_H2b     AATGCCCCAA CTAAATACTA CCGTATGGCC CA-CCATAAT TACCCCCA-T ACTCCTTACA CTATTCCTCA 138
Consensus  AATGCCACAG CTAAATACCA CTGTATGACC TA-CTATCAT CACCCCAA-T ACTCCTCACG TTATTTCTCA

n133_H2b   TCACCCAACT AAAAATACTA AACACACACT GCCATCTGCC CACCTCACCA AAATTTATTA AAATAAAAAA 210
n133_hg18  TCACCCAACT AAAAATACTA AACACACACT GCCATCTGCC CACCTCACCA AAATTTATTA AAATAAAAAA 208
mt_H2b     TCACCCAACT AAAAATATTA AACACAAACT ACCACCTACC TCCCTCACCA AAGCCCATAA AAATAAAAAA 208
Consensus  TCACCCAACT AAAAATACTA AACACACACT GCCATCTGCC CACCTCACCA AAATTTATTA AAATAAAAAA

n133_H2b   CTACAGTAAG CCCTGAGAAC CAAAATGAAC GAAAAATTTA TTCGCTTCAT TCATTACCCC TACAGTACTA 280
n133_hg18  CTACAGTAAG CCCTGAGAAC CAAAATGAAC G-AAAATTTA TTCGCTTCAT TCATTACCCC TACAGTACTA 277
mt_H2b     TTATAACAAA CCCTGAGAAC CAAAATGAAC G-AAAATCTG TTCGCTTCAT TCATTGCCCC CACAATCCTA 277
Consensus  CTACAGTAAG CCCTGAGAAC CAAAATGAAC G-AAAATTTA TTCGCTTCAT TCATTACCCC TACAGTACTA

n133_H2b   GGCCTACCCG CCACAGTACC AATCATCCTA TTTCCCCCCT CTACTGGTCC CAACCTCCAA ATACCTCATC 350
n133_hg18  GGCCTACCCG CCACAGTACC AATCATCCTA TTT-CCCCCC TTACTGGTCC CAACCTCCAA ATACCTCATC 346
mt_H2b     GGCCTACCCG CCGCAGTACT GATCATTCTA TTT-CCCCCT CTATTGATCC CCACCTCCAA ATATCTCATC 346
Consensus  GGCCTACCCG CCACAGTACC AATCATCCTA TTT-CCCCCT CTACTGGTCC CAACCTCCAA ATACCTCATC

n133_H2b   AACAACCGAC TAATCACCAC TCAACAATGA CTACTTCAAC TCACCTTAAA ACAAATAATA ACGATACATA 420
n133_hg18  AACAACCGAC TAATCACCAC TCAACAATGA CTACTTCAAC TCACCTTAAA ACAAATAATA ACGATACATA 416
mt_H2b     AACAACCGAC TAATCACCAC CCAACAATGA CTAATCAAAC TAACCTCAAA ACAAATGATA ACCATACACA 416
Consensus  AACAACCGAC TAATCACCAC TCAACAATGA CTACTTCAAC TCACCTTAAA ACAAATAATA ACGATACATA

n133_H2b   ACATTAAGGG ACGAACCTGG TCCCTTATAC TAATTTCCCT GATTATTTTT ATTGCCACAA CTAATCTCCT 490
n133_hg18  ACATTAAGGG ACGAACCTGG TCCCTTATAC TAATTTCCCT GATTATTTTT ATTGCCACAA CTAATCTCCT 486
mt_H2b     ACACTAAAGG ACGAACCTGA TCTCTTATAC TAGTATCCTT AATCATTTTT ATTGCCACAA CTAACCTCCT 486
Consensus  ACATTAAGGG ACGAACCTGG TCCCTTATAC TAATTTCCCT GATTATTTTT ATTGCCACAA CTAATCTCCT

n133_H2b   CGGACTCTTG CCCCACTCAT TTACACCAAT CAC 523
n133_hg18  CGGACTCTTG CCCCACTCAT TTACACCAAT CAC 519
mt_H2b     CGGACTCCTG CCTCACTCAT TTACACCAAC CAC 519
Consensus  CGGACTCTTG CCCCACTCAT TTACACCAAT CAC
```

NumtS 139

```
                                   20                    40                    60
n139_H2b   ACCAACCTCC GAGAAATCAT CCTCCCGCTC GGGAGTACTA CTGTCTTCGC TCCGGGCCCA TAACACTTGG  70
n139_hg18  ACCAACCTCC GAGAAATCAT CCTCCCGCTC GGGAGTACTA CTGTCTTCGC TCCGGGCCCA TAACACTTGG  70
mt_H2b     ACCATCCTCC GTGAAATCAA TATCCCGCAC AAGAGTGCTA CTCTCCTCGC TCCGGGCCCA TAACACTTGG  70
Consensus  ACCAACCTCC GAGAAATCAT CCTCCCGCTC GGGAGTACTA CTGTCTTCGC TCCGGGCCCA TAACACTTGG
Conservation (100% / 0%)

                     80                    100                   120                   140
n139_H2b   GGGTGACAAT TCTGAAACTA TACCTGGTAT CTGGTTCTTA CTTCAGGGCC ATAAAACTAA GATCGCCCAC  140
n139_hg18  GGGTGACAAT TCTGAAACTA TACCTGGTAT CTGGTTCTTA CTTCAGGGCC ATAAAACTAA GATCGCCCAC  140
mt_H2b     GGGTAGCTAA AGTG-AACTG TATCCGACAT CTGGTTCCTA CTTCAGGGTC ATAAAGCCTA AATAGCCCAC  139
Consensus  GGGTGACAAT TCTGAAACTA TACCTGGTAT CTGGTTCTTA CTTCAGGGCC ATAAAACTAA GATCGCCCAC
Conservation (100% / 0%)

                                  160
n139_H2b   ACTCTCCCCT TAAATAAGAC ATCTCGATG  169
n139_hg18  ACTCTCCCCT TAAATAAGAC ATCTCGATG  169
mt_H2b     ACGTTCCCCT TAAATAAGAC ATCACGATG  168
Consensus  ACTCTCCCCT TAAATAAGAC ATCTCGATG
Conservation (100% / 0%)
```

NumtS 151

```
                                     20                   40                   60
n151_H2b    ACCTGAGTTG TAAAAAACTC CAGTTGATAC AAAATAAACT ACGAAAGTGG CTTTAACATA TCTGAACACG  70
n151_hg18   ACCTGAGTTG TAAAAAACTC CAGTTGATAC AAAATAAACT ACGAAAGTGG CTTTAACATA TCTGAACACA  70
mt_H2b      ACCTGAGTTG TAAAAAACTC CAGTTGACAC AAAATAGACT ACGAAAGTGG CTTTAACATA TCTGAACACA  70
Consensus   ACCTGAGTTG TAAAAAACTC CAGTTGATAC AAAATAAACT ACGAAAGTGG CTTTAACATA TCTGAACACA
Conservation (100% / 0%)

                    80                  100                  120                  140
n151_H2b    CAATAGCTAA GACCCAAACT GGGATTAGAT ACCCCACTAT GCTTAGCCCT AAACCTCAAC AGTTAAATCA  140
n151_hg18   CAATAGCTAA GACCCAAACT GGGATTAGAT ACCCCACTAT GCTTAGCCCT AAACCTCAAC AGTTAAATCA  140
mt_H2b      CAATAGCTAA GACCCAAACT GGGATTAGAT ACCCCACTAT GCTTAGCCCT AAACCTCAAC AGTTAAATCA  140
Consensus   CAATAGCTAA GACCCAAACT GGGATTAGAT ACCCCACTAT GCTTAGCCCT AAACCTCAAC AGTTAAATCA
Conservation (100% / 0%)

                                    160                  180                  200
n151_H2b    ACAAAACTGC TCGCCAGAAC ACTACGAGCC ACAGCTTAAA ACTCAAAGGA CCTGGCGGTG CTTCATATCC  210
n151_hg18   ACAAAACTGC TCGCCAGAAC ACTACGAGCC ACAGCTTAAA ACTCAAAGGA CCTGGCGGTG CTTCATATCC  210
mt_H2b      ACAAAACTGC TCGCCAGAAC ACTACGAGCC ACAGCTTAAA ACTCAAAGGA CCTGGCGGTG CTTCATATCC  210
Consensus   ACAAAACTGC TCGCCAGAAC ACTACGAGCC ACAGCTTAAA ACTCAAAGGA CCTGGCGGTG CTTCATATCC
Conservation (100% / 0%)

                   220                  240
n151_H2b    CTCTAGAGGA GCCTGTTCTG TAATCGATAA ACCCCGATCA ACCTCA  256
n151_hg18   CTCTAGAGGA GCCTGTTCTG TAATCGATAA ACCCCGATCA ACCTCA  256
mt_H2b      CTCTAGAGGA GCCTGTTCTG TAATCGATAA ACCCCGATCA ACCTCA  256
Consensus   CTCTAGAGGA GCCTGTTCTG TAATCGATAA ACCCCGATCA ACCTCA
Conservation (100% / 0%)
```

NumtS 153

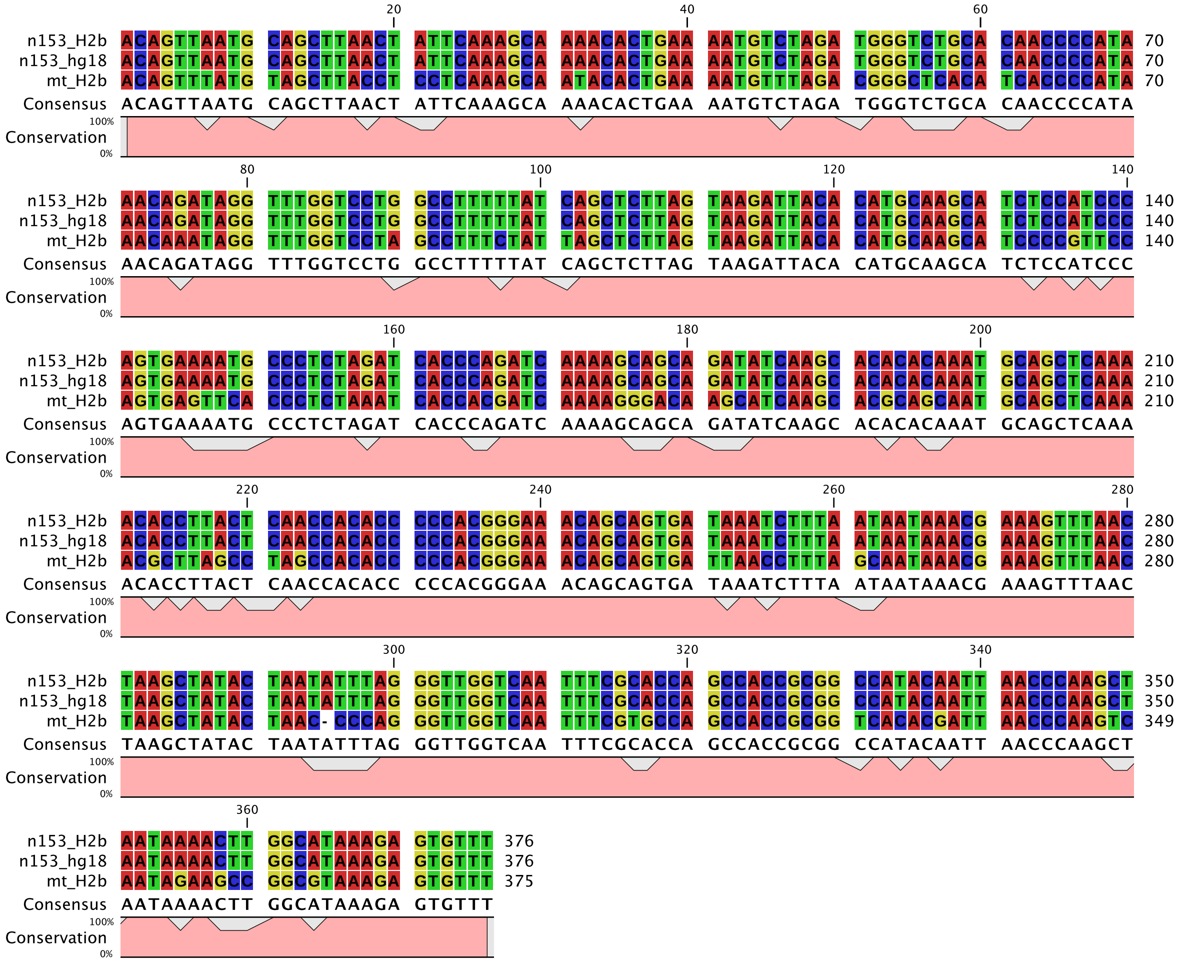
n153_H2b ACAGTTAATG CAGCTTAACT ATTCAAAGCA AAACACTGAA AATGTCTAGA TGGGTCTGCA CAACCCCATA 70
n153_hg18 ACAGTTAATG CAGCTTAACT ATTCAAAGCA AAACACTGAA AATGTCTAGA TGGGTCTGCA CAACCCCATA 70
mt_H2b ACAGTTTATG TAGCTTACCT CCTCAAAGCA ATACACTGAA AATGTTTAGA CGGGCTCACA TCACCCCATA 70
Consensus ACAGTTAATG CAGCTTAACT ATTCAAAGCA AAACACTGAA AATGTCTAGA TGGGTCTGCA CAACCCCATA
Conservation
n153_H2b AACAGATAGG TTTGGTCCTG GCCTTTTTAT CAGCTCTTAG TAAGATTACA CATGCAAGCA TCTCCATCCC 140
n153_hg18 AACAGATAGG TTTGGTCCTG GCCTTTTTAT CAGCTCTTAG TAAGATTACA CATGCAAGCA TCTCCATCCC 140
mt_H2b AACAAATAGG TTTGGTCCTA GCCTTTCTAT TAGCTCTTAG TAAGATTACA CATGCAAGCA TCCCCGTTCC 140
Consensus AACAGATAGG TTTGGTCCTG GCCTTTTTAT CAGCTCTTAG TAAGATTACA CATGCAAGCA TCTCCATCCC
Conservation
n153_H2b AGTGAAAATG CCCTCTAGAT CACCCAGATC AAAAGCAGCA GATATCAAGC ACACACAAAT GCAGCTCAAA 210
n153_hg18 AGTGAAAATG CCCTCTAGAT CACCCAGATC AAAAGCAGCA GATATCAAGC ACACACAAAT GCAGCTCAAA 210
mt_H2b AGTGAGTTCA CCCTCTAAAT CACCACGATC AAAAGGGACA AGCATCAAGC ACGCAGCAAT GCAGCTCAAA 210
Consensus AGTGAAAATG CCCTCTAGAT CACCCAGATC AAAAGCAGCA GATATCAAGC ACACACAAAT GCAGCTCAAA
Conservation
n153_H2b ACACCTTACT CAACCACACC CCCACGGGAA ACAGCAGTGA TAAATCTTTA ATAATAAACG AAAGTTTAAC 280
n153_hg18 ACACCTTACT CAACCACACC CCCACGGGAA ACAGCAGTGA TAAATCTTTA ATAATAAACG AAAGTTTAAC 280
mt_H2b ACGCTTAGCC TAGCCACACC CCCACGGGAA ACAGCAGTGA TTAACCTTTA GCAATAAACG AAAGTTTAAC 280
Consensus ACACCTTACT CAACCACACC CCCACGGGAA ACAGCAGTGA TAAATCTTTA ATAATAAACG AAAGTTTAAC
Conservation
n153_H2b TAAGCTATAC TAATATTTAG GGTTGGTCAA TTTCGCACCA GCCACCGCGG CCATACAATT AACCCAAGCT 350
n153_hg18 TAAGCTATAC TAATATTTAG GGTTGGTCAA TTTCGCACCA GCCACCGCGG CCATACAATT AACCCAAGCT 350
mt_H2b TAAGCTATAC TAAC-CCCAG GGTTGGTCAA TTTCGTGCCA GCCACCGCGG TCACACGATT AACCCAAGTC 349
Consensus TAAGCTATAC TAATATTTAG GGTTGGTCAA TTTCGCACCA GCCACCGCGG CCATACAATT AACCCAAGCT
Conservation
n153_H2b AATAAAACTT GGCATAAAGA GTGTTT 376
n153_hg18 AATAAAACTT GGCATAAAGA GTGTTT 376
mt_H2b AATAGAAGCC GGCGTAAAGA GTGTTT 375
Consensus AATAAAACTT GGCATAAAGA GTGTTT
Conservation

NumtS 157

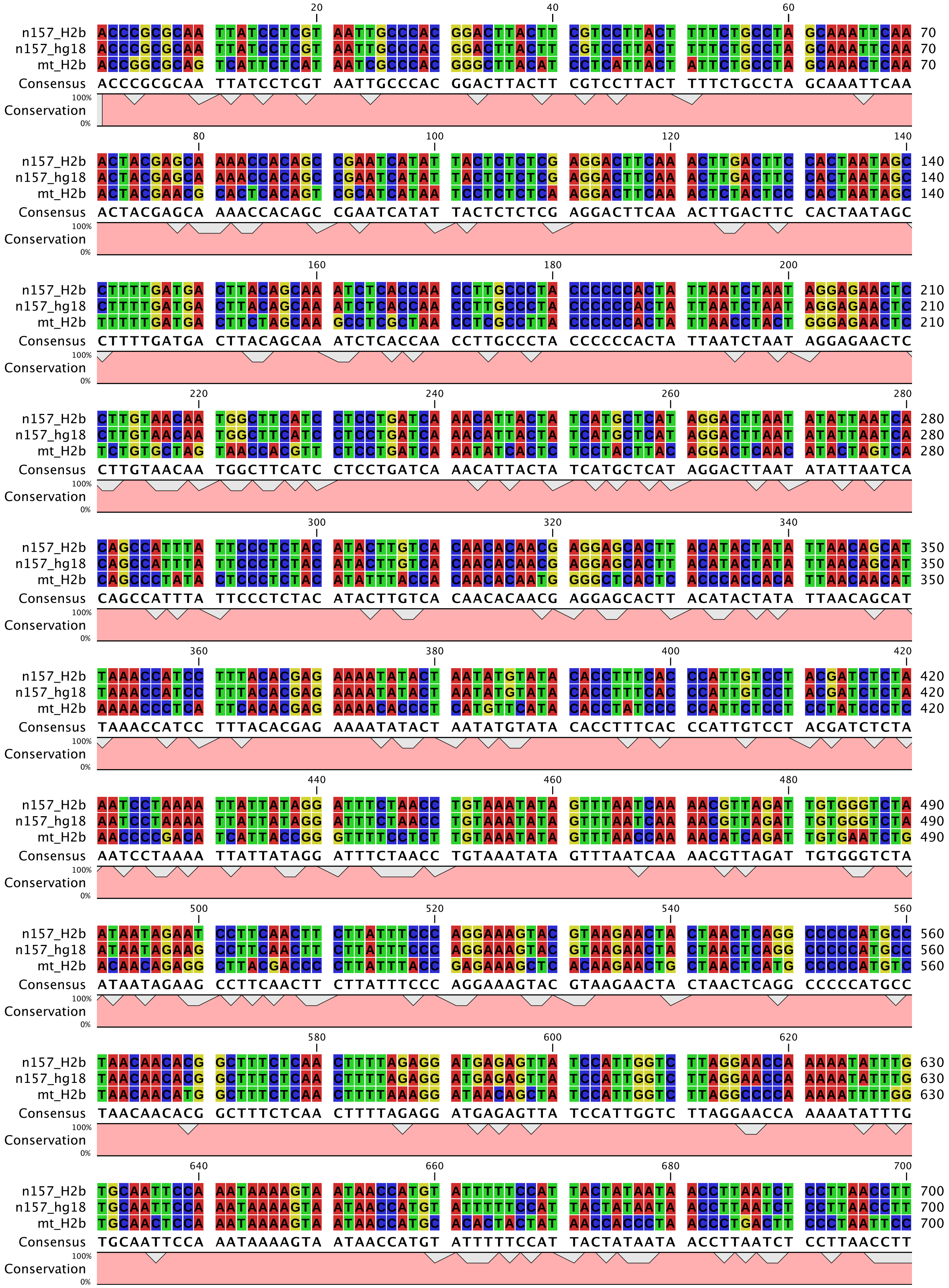
20 40 60
n157_H2b ACCCGCGCAA TTATCCTCGT AATTGCCCAC GGACTTACTT CGTCCTTACT TTTCTGCCTA GCAAATTCAA 70
n157_hg18 ACCCGCGCAA TTATCCTCGT AATTGCCCAC GGACTTACTT CGTCCTTACT TTTCTGCCTA GCAAATTCAA 70
mt_H2b ACCGGCGCAG TCATTCTCAT AATCGCCCAC GGGCTTACAT CCTCATTACT ATTCTGCCTA GCAAACTCAA 70
Consensus ACCCGCGCAA TTATCCTCGT AATTGCCCAC GGACTTACTT CGTCCTTACT TTTCTGCCTA GCAAATTCAA
Conservation 100% 0%
80 100 120 140
n157_H2b ACTACGAGCA AAACCACAGC CGAATCATAT TACTCTCTCG AGGACTTCAA ACTTGACTTC CACTAATAGC 140
n157_hg18 ACTACGAGCA AAACCACAGC CGAATCATAT TACTCTCTCG AGGACTTCAA ACTTGACTTC CACTAATAGC 140
mt_H2b ACTACGAACG CACTCACAGT CGCATCATAA TCCTCTCTCA AGGACTTCAA ACTCTACTCC CACTAATAGC 140
Consensus ACTACGAGCA AAACCACAGC CGAATCATAT TACTCTCTCG AGGACTTCAA ACTTGACTTC CACTAATAGC
Conservation 100% 0%
160 180 200
n157_H2b CTTTTGATGA CTTACAGCAA ATCTCACCAA CCTTGCCCTA CCCCCCACTA TTAATCTAAT AGGAGAACTC 210
n157_hg18 CTTTTGATGA CTTACAGCAA ATCTCACCAA CCTTGCCCTA CCCCCCACTA TTAATCTAAT AGGAGAACTC 210
mt_H2b TTTTTGATGA CTTCTAGCAA GCCTCGCTAA CCTCGCCTTA CCCCCCACTA TTAACCTACT GGGAGAACTC 210
Consensus CTTTTGATGA CTTACAGCAA ATCTCACCAA CCTTGCCCTA CCCCCCACTA TTAATCTAAT AGGAGAACTC
Conservation 100% 0%
220 240 260 280
n157_H2b CTTGTAACAA TGGCTTCATC CTCCTGATCA AACATTACTA TCATGCTCAT AGGACTTAAT ATATTAATCA 280
n157_hg18 CTTGTAACAA TGGCTTCATC CTCCTGATCA AACATTACTA TCATGCTCAT AGGACTTAAT ATATTAATCA 280
mt_H2b TCTGTGCTAG TAACCACGTT CTCCTGATCA AATATCACTC TCCTACTTAC AGGACTCAAC ATACTAGTCA 280
Consensus CTTGTAACAA TGGCTTCATC CTCCTGATCA AACATTACTA TCATGCTCAT AGGACTTAAT ATATTAATCA
Conservation 100% 0%
300 320 340
n157_H2b CAGCCATTTA TTCCCTCTAC ATACTTGTCA CAACACAACG AGGAGCACTT ACATACTATA TTAACAGCAT 350
n157_hg18 CAGCCATTTA TTCCCTCTAC ATACTTGTCA CAACACAACG AGGAGCACTT ACATACTATA TTAACAGCAT 350
mt_H2b CAGCCCTATA CTCCCTCTAC ATATTTACCA CAACACAATG GGGCTCACTC ACCCACCACA TTAACAACAT 350
Consensus CAGCCATTTA TTCCCTCTAC ATACTTGTCA CAACACAACG AGGAGCACTT ACATACTATA TTAACAGCAT
Conservation 100% 0%
360 380 400 420
n157_H2b TAAACCATCC TTTACACGAG AAAATATACT AATATGTATA CACCTTTCAC CCATTGTCCT ACGATCTCTA 420
n157_hg18 TAAACCATCC TTTACACGAG AAAATATACT AATATGTATA CACCTTTCAC CCATTGTCCT ACGATCTCTA 420
mt_H2b AAAACCCTCA TTCACACGAG AAAACACCCT CATGTTCATA CACCTATCCC CCATTCTCCT CCTATCCCTC 420
Consensus TAAACCATCC TTTACACGAG AAAATATACT AATATGTATA CACCTTTCAC CCATTGTCCT ACGATCTCTA
Conservation 100% 0%
440 460 480
n157_H2b AATCCTAAAA TTATTATAGG ATTTCTAACC TGTAAATATA GTTTAATCAA AACGTTAGAT TGTGGGTCTA 490
n157_hg18 AATCCTAAAA TTATTATAGG ATTTCTAACC TGTAAATATA GTTTAATCAA AACGTTAGAT TGTGGGTCTA 490
mt_H2b AACCCCGACA TCATTACCGG GTTTTCCTCT TGTAAATATA GTTTAACCAA AACATCAGAT TGTGAATCTG 490
Consensus AATCCTAAAA TTATTATAGG ATTTCTAACC TGTAAATATA GTTTAATCAA AACGTTAGAT TGTGGGTCTA
Conservation 100% 0%
500 520 540 560
n157_H2b ATAATAGAAT CCTTCAACTT CTTATTTCCC AGGAAAGTAC GTAAGAACTA CTAACTCAGG CCCCCATGCC 560
n157_hg18 ATAATAGAAG CCTTCAACTT CTTATTTCCC AGGAAAGTAC GTAAGAACTA CTAACTCAGG CCCCCATGCC 560
mt_H2b ACAACAGAGG CTTACGACCC CTTATTTACC GAGAAAGCTC ACAAGAACTG CTAACTCATG CCCCCATGTC 560
Consensus ATAATAGAAG CCTTCAACTT CTTATTTCCC AGGAAAGTAC GTAAGAACTA CTAACTCAGG CCCCCATGCC
Conservation 100% 0%
580 600 620
n157_H2b TAACAACACG GCTTTCTCAA CTTTTAGAGG ATGAGAGTTA TCCATTGGTC TTAGGAACCA AAAATATTTG 630
n157_hg18 TAACAACACG GCTTTCTCAA CTTTTAGAGG ATGAGAGTTA TCCATTGGTC TTAGGAACCA AAAATATTTG 630
mt_H2b TAACAACATG GCTTTCTCAA CTTTTAAAGG ATAACAGCTA TCCATTGGTC TTAGGCCCCA AAAATTTTGG 630
Consensus TAACAACACG GCTTTCTCAA CTTTTAGAGG ATGAGAGTTA TCCATTGGTC TTAGGAACCA AAAATATTTG
Conservation 100% 0%
640 660 680 700
n157_H2b TGCAATTCCA AATAAAAGTA ATAACCATGT ATTTTTCCAT TACTATAATA ACCTTAATCT CCTTAACCTT 700
n157_hg18 TGCAATTCCA AATAAAAGTA ATAACCATGT ATTTTTCCAT TACTATAATA ACCTTAATCT CCTTAACCTT 700
mt_H2b TGCAACTCCA AATAAAAGTA ATAACCATGC ACACTACTAT AACCACCCTA ACCCTGACTT CCCTAATTCC 700
Consensus TGCAATTCCA AATAAAAGTA ATAACCATGT ATTTTTCCAT TACTATAATA ACCTTAATCT CCTTAACCTT
Conservation 100% 0%

```
                        720                       740                       760
n157_H2b   ACCAATT--- ---------- ---------- ---------- ---------- ---------- ----------  707
n157_hg18  ACCAATTATT ATTGCCTTCA CCAACCCTGG TAAGAAGAAC TCGTATCCAT ATTACGTAAA AAGC--TATC  768
mt_H2b     CCCCATCCTT ACCACCCTCG TTAACCCTAA CAAAAAAAAC TCATACCCCC ATTATGTAAA ATCCATTGTC  770
Consensus  ACCAATTNTT ANNNCCNTCN NNAACCCTNN NAANAANAAC TCNTANCCNN ATTANGTAAA ANNC--TNTC
Conservation 100% 0%

              780                       800                       820                  840
n157_H2b   ---------- ---------- ---------- ---------- ---------- ---------- ----------  707
n157_hg18  TCAAGCACTT TCGCCATTAG CCTCCTTCCG GCAACAATAT TTATGTGTAC AGACCAAGAA GCCATCATCT  838
mt_H2b     GCATCCACCT TTATTATCAG TCTCTTCCCC ACAACAATAT TCATGTGCCT AGACCAAGAA GTTATTATCT  840
Consensus  NCANNCACNT TNNNNATNAG NCTCNTNCCN NCAACAATAT TNATGTGNNN AGACCAAGAA GNNATNATCT
Conservation 100% 0%

                        860                       880                       900
n157_H2b   ---------- ---------- ---------- ---------- ---------- ---------- ----------  707
n157_hg18  CAAACTGATA TTGAATAACA ATCCAAACCC CAAAACTCTC ATTAAGCTTC AAACTAGACT ACTTCCCA--  906
mt_H2b     CGAACTGACA CTGAGCCACA ACCCAAACAA CCCAGCTCTC CCTAAGCTTC AAACTAGACT ACTTCTCCAT  910
Consensus  CNAACTGANA NTGANNNACA ANCCAAACNN CNNANCTCTC NNTAAGCTTC AAACTAGACT ACTTCNCN--
Conservation 100% 0%

              920                       940                       960                  980
n157_H2b   ---------- ---------- ---------- ---------- ---------- ---------- ----------  707
n157_hg18  AATGTTTATT CCAGTAGCAC TATTTATCAC TTGATCTATT ATAGAATTCT CA-TATGGTA TATAAAGTCA  975
mt_H2b     AATATTCATC CCTGTAGCAT TGTTCGTTAC ATGGTCCATC ATAGAATTCT CACTGTGATA TATAAACTCA  980
Consensus  AATNTTNATN CCNGTAGCAN TNTTNNTNAC NTGNTCNATN ATAGAATTCT CA-TNTGNTA TATAAANTCA
Conservation 100% 0%

                        1.000                     1.020                     1.040
n157_H2b   ---------- ---------- ---------- ---------- ---------- ---------- ----------  707
n157_hg18  GATCCAAACA TTAATCAGTT TTTCCAATAT CTACACATCT TCCTCACCAC AATATTAATC CTAGTTACCG  1045
mt_H2b     GACCCAAACA TTAATCAGTT CTTCAAATAT CTACTCATCT TCCTAATTAC CATACTAATC TTAGTTACCG  1050
Consensus  GANCCAAACA TTAATCAGTT NTTCNAATAT CTACNCATCT TCCTNANNAC NATANTAATC NTAGTTACCG
Conservation 100% 0%

              1.060                     1.080                     1.100                1.120
n157_H2b   ---------- ---------- ---------- ---------- ---------- ---------- ----------  707
n157_hg18  CCAACAACTC TTTCCAACTT TTTATTGGAT GAGAAGGCGT AGGAATTATA TCTTTTCTAT TAATTGGCTG  1115
mt_H2b     CTAACAACCT ATTCCAACTG TTCATCGGCT GAGAGGGCGT AGGAATTATA TCCTTCTTGC TCATCAGTTG  1120
Consensus  CNAACAACNN NTTCCAACTN TTNATNGGNT GAGANGGCGT AGGAATTATA TCNTTNNTNN TNATNNGNTG
Conservation 100% 0%

                        1.140                     1.160                     1.180
n157_H2b   ---------- ---------- ---------- ---------- ---------- ---------- ----------  707
n157_hg18  ATGGCATGGC CGAGCAGATG CTCACACGGC AGCCCTCCAA GCAATTCTAT ATAATTGCAT TGGTGATCTT  1185
mt_H2b     ATGATACGCC CGAGCAGATG CCAACACAGC AGCCATTCAA GCAATCCTAT ACAACCGTAT CGGCGATATC  1190
Consensus  ATGNNANGNC CGAGCAGATG CNNACACNGC AGCCNTNCAA GCAATNCTAT ANAANNGNAT NGGNGATNTN
Conservation 100% 0%

              1.200                     1.220                     1.240                1.260
n157_H2b   ---------- ---------- ---------- ---------- ---------- ---------- ----------  707
n157_hg18  GGCTTTATTT TAGCTATAGC ATGATTCCTG TCATCCTCCA ATACATGAGA GCCTCAACAA ATACTGATCC  1255
mt_H2b     GGTTTCATCC TGCCCTTAGC ATGATTTATC CTACACTCCA ACTCATGAGA CCCACAACAA ATAGCCCTTC  1260
Consensus  GGNTTNATNN TNGCNNTAGC ATGATTNNTN NNANNCTCCA ANNCATGAGA NCCNCAACAA ATANNNNTNC
Conservation 100% 0%

                        1.280                     1.300                     1.320
n157_H2b   ---------- ---------- ---------- ---------- ---------- ---------- ----------  707
n157_hg18  TAAACACT-A CCCGGATCTT CTTCCATTAA TTGGTTTTCT CTTAGCAGCA GCAGGAAAAT CAGCCCAACT  1324
mt_H2b     TAAACGCTAA TCCAAGCCTC ACCCCACTAC TAGGCCTCCT CCTAGCAGCA GCAGGCAAAT CAGCCCAATT  1330
Consensus  TAAACNCT-A NCCNNNNCTN NNNCCANTAN TNGGNNTNCT CNTAGCAGCA GCAGGNAAAT CAGCCCAANT
Conservation 100% 0%

              1.340                     1.360                     1.380                1.400
n157_H2b   ---------- ---------- ---------- ---------- ---------- ---------- ----------  707
n157_hg18  TGGCCTCCAC CCGTGACTTC CTTCCGCGAT GGAAGGCCCA ACCCCAGTCT CAGCCCTTCT TCACTCTAGC  1394
mt_H2b     AGGTCTCCAC CCCTGACTCC CCTCAGCCAT AGAAGGCCCC ACCCCAGTCT CAGCCCTACT CCACTCAAGC  1400
Consensus  NGGNCTCCAC CCNTGACTNC CNTCNGCNAT NGAAGGCCCN ACCCCAGTCT CAGCCCTNCT NCACTCNAGC
Conservation 100% 0%
```

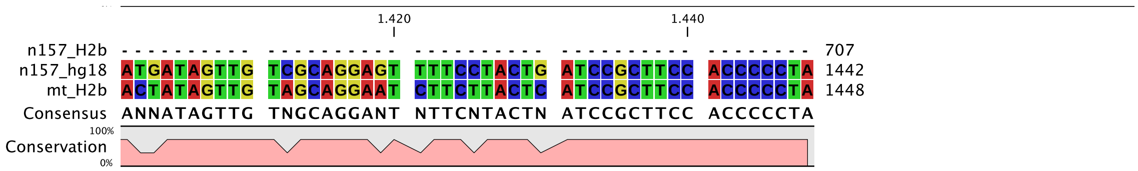
1.420
1.440
n157_H2b ---------- ---------- ---------- ---------- -------- 707
n157_hg18 ATGATAGTTG TCGCAGGAGT TTTCCTACTG ATCCGCTTCC ACCCCCTA 1442
mt_H2b ACTATAGTTG TAGCAGGAAT CTTCTTACTC ATCCGCTTCC ACCCCCTA 1448
Consensus ANNATAGTTG TNGCAGGANT NTTCNTACTN ATCCGCTTCC ACCCCCTA
100%
Conservation
0%

NumtS 169

```
                                    20                    40                    60
n169_H2b    ATGCGCTAAC CCCACTAAAA CACTTACCAA GACCTCAACC CCTGACCCCC ATGCCTCAGG ATACTCCTCA  70
n169_hg18   AT-CGCTAAC CCCACTAAAA CACTTACCAA GACCTCAACC CCTGACCCCC ATGCCTCAGG ATACTCCTCA  69
mt_H2b      AT-CGCTAAC CCCACTAAAA CACTCACCAA GACCTCAACC CCTGACCCCC ATGCCTCAGG ATACTCCTCA  69
Consensus   AT-CGCTAAC CCCACTAAAA CACTTACCAA GACCTCAACC CCTGACCCCC ATGCCTCAGG ATACTCCTCA
            100%
Conservation
            0%

                      80                   100                   120                   140
n169_H2b    ATAGCCATCG CTGTAGTGTA CCCAAAGACA ACCCTTATTC CCCCCAAATA AATT-TAAAA ACCATTAAAC  139
n169_hg18   ATAGCCATCG CTGTAGTGTA CCCAAAGACA ACCCTTATTC CCCCCAAATA AATT-TAAAA ACCATTAAAC  138
mt_H2b      ATAGCCATCG CTGTAGTATA TCCAAAGACA ACCATCATTC CCCCTAAATA AATTAAAAAA ACTATTAAAC  139
Consensus   ATAGCCATCG CTGTAGTGTA CCCAAAGACA ACCCTTATTC CCCCCAAATA AATT-TAAAA ACCATTAAAC
            100%
Conservation
            0%

n169_H2b    CCATGTAACC TCCCCCA  156
n169_hg18   CCATGTAACC TCCCCCA  155
mt_H2b      CCATATAACC TCCCCCA  156
Consensus   CCATGTAACC TCCCCCA
            100%
Conservation
            0%
```

NumtS C

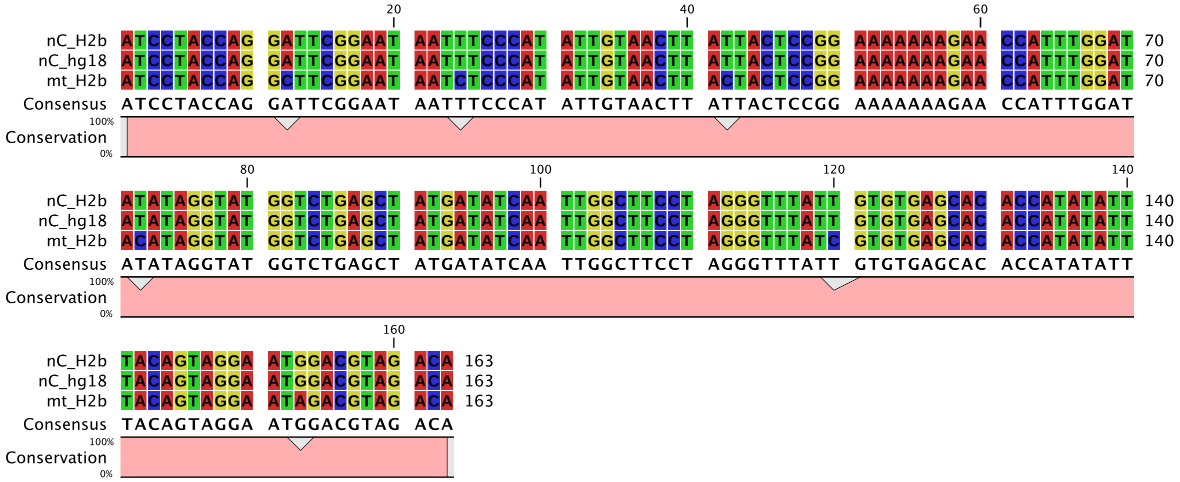

20 40 60
nC_H2b ATCCTACCAG GATTCGGAAT AATTTCCCAT ATTGTAACTT ATTACTCCGG AAAAAAAGAA CCATTTGGAT 70
nC_hg18 ATCCTACCAG GATTCGGAAT AATTTCCCAT ATTGTAACTT ATTACTCCGG AAAAAAAGAA CCATTTGGAT 70
mt_H2b ATCCTACCAG GCTTCGGAAT AATCTCCCAT ATTGTAACTT ACTACTCCGG AAAAAAAGAA CCATTTGGAT 70
Consensus ATCCTACCAG GATTCGGAAT AATTTCCCAT ATTGTAACTT ATTACTCCGG AAAAAAAGAA CCATTTGGAT
100%
Conservation
0%
80 100 120 140
nC_H2b ATATAGGTAT GGTCTGAGCT ATGATATCAA TTGGCTTCCT AGGGTTTATT GTGTGAGCAC ACCATATATT 140
nC_hg18 ATATAGGTAT GGTCTGAGCT ATGATATCAA TTGGCTTCCT AGGGTTTATT GTGTGAGCAC ACCATATATT 140
mt_H2b ACATAGGTAT GGTCTGAGCT ATGATATCAA TTGGCTTCCT AGGGTTTATC GTGTGAGCAC ACCATATATT 140
Consensus ATATAGGTAT GGTCTGAGCT ATGATATCAA TTGGCTTCCT AGGGTTTATT GTGTGAGCAC ACCATATATT
100%
Conservation
0%
160
nC_H2b TACAGTAGGA ATGGACGTAG ACA 163
nC_hg18 TACAGTAGGA ATGGACGTAG ACA 163
mt_H2b TACAGTAGGA ATAGACGTAG ACA 163
Consensus TACAGTAGGA ATGGACGTAG ACA
100%
Conservation
0%
